# Neurobehavioral dysfunction in non-alcoholic steatohepatitis is associated with hyperammonemia, gut dysbiosis, and metabolic and functional brain regional deficits

**DOI:** 10.1371/journal.pone.0223019

**Published:** 2019-09-20

**Authors:** Sara G. Higarza, Silvia Arboleya, Miguel Gueimonde, Eneritz Gómez-Lázaro, Jorge L. Arias, Natalia Arias

**Affiliations:** 1 Institute of Neurosciences of the Principality of Asturias (INEUROPA), Asturias, Spain; 2 Laboratory of Neuroscience, Department of Psychology, University of Oviedo, Oviedo, Asturias, Spain; 3 Department of Microbiology and Biochemistry of Dairy Products, Institute of Dairy Products of the Principality of Asturias (IPLA-CSIC), Asturias, Spain; 4 Department of Basic Psychological Processes and their Development, Basque Country University, San Sebastián, Basque Country, Spain; 5 Department of Basic and Clinical Neuroscience, Institute of Psychiatry, Psychology and Neuroscience, King's College London, London, England, United Kingdom; University of Leicester, UNITED KINGDOM

## Abstract

Non-alcoholic steatohepatitis (NASH) is one of the most prevalent diseases worldwide. While it has been suggested to cause nervous impairment, its neurophysiological basis remains unknown. Therefore, the aim of this study is to unravel the effects of NASH, through the interrelationship of liver, gut microbiota, and nervous system, on the brain and human behavior. To this end, 40 Sprague-Dawley rats were divided into a control group that received normal chow and a NASH group that received a high-fat, high-cholesterol diet. Our results show that 14 weeks of the high-fat, high-cholesterol diet induced clinical conditions such as NASH, including steatosis and increased levels of ammonia. Rats in the NASH group also demonstrated evidence of gut dysbiosis and decreased levels of short-chain fatty acids in the gut. This may explain the deficits in cognitive ability observed in the NASH group, including their depressive-like behavior and short-term memory impairment characterized in part by deficits in social recognition and prefrontal cortex-dependent spatial working memory. We also reported the impact of this NASH-like condition on metabolic and functional processes. Brain tissue demonstrated lower levels of metabolic brain activity in the prefrontal cortex, thalamus, hippocampus, amygdala, and mammillary bodies, accompanied by a decrease in dopamine levels in the prefrontal cortex and cerebellum and a decrease in noradrenalin in the striatum. In this article, we emphasize the important role of ammonia and gut-derived bacterial toxins in liver-gut-brain neurodegeneration and discuss the metabolic and functional brain regional deficits and behavioral impairments in NASH.

## Introduction

Non-Alcoholic Fatty Liver disease (NAFLD) is one of the most prevalent diseases in the world. It is defined by the presence of steatosis in a minimum of 5% of hepatocytes, in the absence of significant alcohol consumption [1]. NAFLD includes a spectrum of conditions, ranging from simple steatosis to steatohepatitis (NASH). NASH is characterized by overt inflammation and occasional fibrosis that may progress to cirrhosis and hepatocellular carcinoma [2]. The factors controlling the progression from NAFLD to NASH remain unclear, but lipotoxicity, oxidative stress, and proinflammatory mediators have been suggested to play a critical role in the development of this disease [3,4].

Various diets have been investigated as potential NASH models, although many of these models do not replicate all the features of the human condition [5]. The high-fat, high-cholesterol animal model reported in this study was previously described in a publication documenting a rat NASH model [6,7] which demonstrated the typical features of NAFLD pathology, including hepato- and splenomegaly, early NASH histopathology, hypercholesterolemia, increased serum liver enzymes, and increased pro-inflammatory cytokines [6,8–10].

Nevertheless, only a few studies have addressed the risk profile with regards to the impact of these diets on brain and cognition. Recent studies have associated fat intake with a higher risk of cognitive decline and dementia [11]. However, Deshpande et al. [12] showed that in early middle-aged Sprague Dawley rats fed a high-fat diet for 8 weeks, the gut microbiota is altered, without any evidence of spatial working memory deficits. There was no evidence of neuroinflammation either, as measured by microglia in the cortex, hippocampus, and hypothalamus. In contrast, several studies have shown hippocampal-dependent memory alterations and neuroinflammation in middle-aged and aged animal models on both high-fat and high-cholesterol diets [13,14]. Therefore, the diet duration, diet composition (in terms of fat, fructose, and carbohydrates), animal model age (young, middle-aged, or aged), and animal model strain are all important factors to consider when interpreting these results.

Understanding the mechanisms by which a high-fat, high-cholesterol diet alters neural environments such as to result in cognitive decline is important to the identification of potential therapeutic targets and the development of treatments. The composition of the gut microbiota has emerged as one of the main potential factors modulating brain function via the liver-gut-brain axis. Predictive analyses of functional microbiota pathways in functional gastrointestinal disorders [15–17] demonstrated dampened activity in pathways involved in the metabolism of propanoate and butanoate, conjugate bases of the short-chain fatty acids (SCFAs) propionate and butyrate [18]. These SCFAs have been shown not only to simulate vagus nerve signaling [19,20] but also to alter levels of neurochemicals such as serotonin [21,22], pointing to their role in gut-brain associated neurodegeneration. The net effect of such SCFAs on NASH-brain associated pathogenesis therefore remains unclear.

Finally, studies in fructose-induced metabolic syndrome-like conditions have demonstrated altered mitochondrial function, crucial in supporting brain function and plasticity [23]. In line with these observations, Mastrocola et al. [24] demonstrated the altered activity of mitochondrial respiratory complexes in hippocampi of mice exposed to high-fructose intake. Thus, changes in the metabolic capacity of neurons associated with the diet administered in this study may be measured through mitochondrial enzymes that catalyze oxygen consumption in cellular respiration, such as cytochrome c oxidase (CCO) [25].

The aim of the present study was to investigate the effects of a high-fat, high-cholesterol (HFHC) diet in a rat NASH model (14 weeks) on the liver-gut-brain axis. We hypothesized that the development of NASH would lead to gut dysbiosis and impaired brain communication by disrupting metabolic processes and neurotransmission. In addition, we evaluated the impact of this diet on behavioral deficits, including motor, emotional, and cognitive aspects. As such, the study’s objective was reached, leading to a better knowledge on the role of the liver-gut-brain axis in NASH.

## Materials and methods

### Procurement of experimental models

A total of 40 male Sprague-Dawley rats were used (220 g at the start of the experiment, 8 weeks old) (Envigo, Blackthorn, United Kingdom) and divided into two groups: NC (control group), which received normal chow, and the NASH group, which was administered a high-fat, high-cholesterol diet (HFHC) for 14 weeks. The NC (Envigo, Blackthorn, United Kingdom; #2914) diet consisted of 13 kcal% from fat and no cholesterol, whereas the HFHC diet (Research Diets, New Brunswick, NJ, USA; #D09052204) contained 65 kcal% from fat, 2 kcal% from cholesterol, and 0.5% cholate. Heebøll et al. [9] showed that the HFHC diet is capable of replicating the histological features of NASH. The high concentration of cholesterol in the diet induces the progression of hepatic steatosis to NASH [26]. The inclusion of a high concentration of cholate further drives the progression to NASH because cholate has been shown to increase the hepatic accumulation of cholesterol [27]. Finally, male rats were used because they are more susceptible to developing hepatic steatosis [28].

All the animals had *ad libitum* access to tap water and were maintained at constant room temperature (22 ± 2 °C), with a relative humidity of 65 ± 5% and an artificial light-dark cycle of 12 h (08:00–20:00/20:00–08:00 h). All behavioral testing was conducted between 08.00 and 13:00 h. The procedures and manipulation of the animals used in this study were carried out according to the Directive (2010/63/EU), Royal Decree 53/2013 of the Ministry of the Presidency related to the protection of animals used for experimentation and other scientific purposes. The Oviedo Animal Experiment Inspectorate approved of the experimental protocol (Permit number: PROAE 25/2018), which abided by the terms of the UK Animals (Scientific Procedures) Act 1986 and were approved by the Kings College London ethics review panel (Permit number: P35785fd7). All efforts were made to minimize suffering.

Experimental animals in both groups were submitted to portal pressure measurements, liver histological evaluation, biochemical plasma determination, fecal microbiota evaluation, and motor function evaluation, and were tested for anxiety-like and depressive-like behaviors, anhedonia, and social short-term memory. To avoid interference between spatial memory procedures, each group (NC and NASH; n/group = 20) was divided into three subgroups. One subgroup was submitted to the evaluation of the spatial reference memory test in the radial arm water maze (RAWM) (n/group = 6). The second subgroup was tested for the same memory, but using a different test, the Barnes maze (n/group = 5). Finally, the third subgroup (n/group = 9) was evaluated on the spatial working memory test, and their brains were used to analyze brain metabolic and functional activity.

### Portal pressure

Portal pressure was measured under anesthesia (2% isofluorane in oxygen) (Fatro Ibérica, Barcelona, Spain) by direct cannulation of the main portal vein (n: NC = 15, NASH = 11).

### Sacrifice

Ninety minutes after the last session of the spatial working memory task, the animals were decapitated. Blood was collected and centrifuged to collect plasma, which was frozen in N-methylbutane (Sigma-Aldrich, St. Louis, MO, USA) and stored at −80 °C. Organs were removed and weighed (n/group = 15). Livers were fixed with a 10% formaldehyde solution (Fisher Scientific, Hampton, VA, USA) and embedded in paraffin (Merck, Darmstadt, Germany). One hemisphere of each brain was dissected into prefrontal cortex, striatum, hippocampus, and cerebellum in order to assess the presence of monoamines. The other hemisphere was rapidly frozen in N-methylbutane (Sigma-Aldrich, St. Louis, MO, USA) and stored at −40 °C for metabolic activity.

### Liver histological examination

Liver histology microscopy was performed in 10 μm thick sections of paraffin-embedded liver tissue previously deparaffinised and stained with Picrosirius red (Sirius red F3BA 0.1% w/vol in saturated picric acid) (Sigma-Aldrich, St. Louis, MO, USA) for 90 min, washed in glacial acetic acid (Probus, Madrid, Spain) and water (5:1000), dehydrated in ethanol (VWR, Radnor, PA, USA), and mounted in Entellan (Merck, Darmstadt, Germany).

### Biochemical plasma determination

Plasma biochemistry was performed using a Cobas Integra II system (Roche Diagnostics, Rotkreuz, Switzerland). A total of 29 animals were analyzed (n: NC = 14 and NASH = 15). Plasma was biochemically assessed for the presence of albumin, ammonia, aspartate aminotransferase (AST), alanine aminotransferase (ALT), total protein, glucose, creatinine, urine, bilirubin, total cholesterol, high density lipoprotein (HDL) cholesterol, low density lipoprotein (LDL) cholesterol, and triglycerides.

### Fecal microbiota exploration

#### Fecal samples collection and processing

Fresh fecal pellets were obtained from each animal (n: NC = 10, NASH = 9) and frozen at -80°C. For the analyses, the samples were melted at RT, weighed, diluted in PBS (1:5 w/v), and homogenized (3 min, full speed) in a Stomacher (VWR, Radnor, PA, USA; Lab Blender #400). Next, 1 mL of the homogenate was centrifuged (10.000 rpm, 15 min), and the fecal supernatant was stored at -20°C in order to use the QIAamp DNA stool kit (Qiagen GmbH, Hilden, Germany), as described elsewhere [29]. The DNA obtained was then stored at -20°C until further analysis.

#### Analysis of fecal microbial groups by 16S rRNA gene profiling

Extracted DNA was used as a template for the amplification of partial 16S rRNA gene sequences by PCR using the primers and conditions described by Milani and co-workers [30]. The amplicons obtained were then sequenced using the MiSeq (Illumina, San Diego, CA, USA) platform at GenProbio s.r.l. (Parma, Italy). The individual reads obtained were filtered, trimmed, and processed [31]. Next, 16S rRNA Operational Taxonomic Units were defined at ≥ 97% sequence homology using the uclust tool developed by Edgar et al. [32]. All reads were classified into the lowest possible taxonomic rank using QIIME and a reference dataset from the SILVA database [33].

#### Analysis of fecal microbial groups by quantitative PCR

To gain further insight into the assessment of the fecal microbiota of the NC and NASH animals, the absolute levels of selected microbial groups, including the *Bacteroides*-group, *Lactobacillus*-group *Clostridium* XIVa-group, *Clostridium* IV-group, *Enterobacteriaceae*, and the genera *Bifidobacterium* and *Akkermansia*, as well as the levels of total bacteria, were determined by quantitative PCR using previously described primers and conditions [34,35].

#### Determination of SCFAs in feces

SCFAs levels were determined in the fecal supernatants by means of gas chromatography, as described by Moris et al. [36]. Briefly, cell free-supernatants (250 μl) from fecal homogenates, prepared as indicated above, were mixed with 100 μl methanol (Merck, Darmstadt, Germany), 50 μl internal standard solution (2-ethylbutyric 1.05 mg/ml) (Sigma-Aldrich, St. Louis, MO, USA), and 50 μl of 20% v/v formic acid (Sigma-Aldrich, St. Louis, MO, USA). The mix was then centrifuged, and the supernatant was used to quantify SCFAs in a system composed of a 6890NGC injection module connected to a flame injection detector (FID) and a mass spectrometry detector (MS, 5973N) (Agilent Technologies, Madrid, Spain).

### Behavioral evaluation

#### Test for assessment of locomotor activity: Rotarod-accelerod test

The test aimed at evaluating motor performance consists of a motor-driven rotating rod that enables the assessment of motor coordination and resistance to fatigue [37]. The accelerating rotarod 7750 by Ugo Basile (Ugo Basile Biological Research Apparatus, Gemonio, Italy) was used to this end to assess the rats’ motor performance (n/group = 15). The procedure was separated into two steps. In the first part, animals were placed in the apparatus, and the speed was maintained constant at 2 rpm for 60 s. In the second part, rats were evaluated for 5 min in the accelerod test session, in which the rotation rate constantly increased until reaching 20 rpm. Latency to fall off the rod (s) and the actual rotation speed (rpm) were recorded.

#### Tests for assessment of anxiety- and depressive-like behavior

Anxiety-like behavior was evaluated in the open field exploratory test, whereas depressive-like behavior was explored through the evaluation of two different aspects, anhedonic state (sucrose preference test) and despair (forced swimming test).

The open field exploratory test provides a unique opportunity to systematically assess the exploration of a novel environment and general locomotor activity while providing an initial screening tool for anxiety-related behavior in rodents. It has been suggested that two factors influence anxiety-like behavior in the open field. The first factor is social isolation resulting from the physical separation from cage mates during the test. The second factor is stress induced by the brightly lit, unprotected, and novel test environment.

The evaluation of anxiety-like behavior was performed in an open field consisting of a square arena (86×86 cm) with a white floor divided into two zones (periphery and center) enclosed by continuous, 12.5-cm-high walls made of black Plexiglass. The arena was lit by two light lamps that provided an illumination density of approximately 1200 lux at the center of the open field. On this test, the zone adjacent to the wall represents a protected field, named the ‘arena periphery’, whereas the other represents an exposed field, named the ‘arena center’. The test was initiated by placing a single rat in the middle of the arena and letting it move freely for 5 min. Animal behavior was recorded with a computerized video-tracking system (EthoVision Pro, Noldus Information Technologies, Wageningen, The Netherlands). Durations spent in the central and peripheral areas were measured.

In contrast, depressive-like behavior was assessed through the evaluation of anhedonic state and despair. In the sucrose preference test, aimed at assessing anhedonia, animals were transferred into single housing units with access to food *ad libitum*. Each rat was provided with two water bottles on either edge of the cage during the 24-h training phase in order to allow them to get used to drinking from two water bottles. Following training, one bottle was randomly switched to another bottle containing a 0.8% sucrose solution, as described previously [38]. After another 24 h, the 0.8% sucrose solution bottle was replaced with water, and drinking was measured for 24 h before one of the two bottles was removed from the cage. The use of a 48-h testing period allowed us to preclude any effects of neophobia, artefactual bias toward one side, and perseveration. Furthermore, it provides information on long-term access to a rewarding stimulus. The consumption of water and sucrose solution was estimated simultaneously in all groups (n/group = 15).

The procedure for the forced swimming test, used to evaluate despair, was previously described by Porsolt et al. [39]. Rats were placed in an individual glass cylinder (40 cm in height x 17 cm in diameter) containing water (30 cm water depth, 25°C). Two swimming sessions were conducted (an initial 10-min pre-test followed 24 h later by a 6-min test). The total duration of climbing, swimming, and immobility was manually scored continuously over the course of the last 4 minutes of the test. Climbing behavior consisted of upward-directed movements of the forepaws along the side of the swim chamber. Swimming behavior consisted of movement (usually horizontal) throughout the swim chamber. Rats were considered immobile when not engaging in any additional activity other than that which was required to keep the rat's head above the water. Behavior was recorded for a second independent analysis by a blinded experimenter, using a computerized video-tracking system (EthoVision Pro, Noldus Information Technologies, Wageningen, The Netherlands) (n/group = 15).

#### Tests for assessment of cognitive function

The animals were tested on the social recognition test to assess their social recognition memory and reaction to novelty. Hippocampal function was determined by measuring spatial reference memory in the radial arm water maze (RAWM) and in the Barnes maze, whereas prefrontal cortex function was evaluated while performing the spatial working memory task in the RAWM.

The animals (NC = 13, NASH = 10) were tested on the social recognition test to assess their social recognition memory and reaction to novelty, as described previously [40]. Sprague Dawley male rats were used as intruders. Before the first trial, an empty chamber was placed in the test cage, which the rat was allowed to spontaneously explore (non-social). During an “initial encounter”, an intruder was placed inside a transparent acrylic chamber with several orifices in its walls. The sessions consisted of five trials lasting 5 min each, separated by 10 min intervals. In the subsequent four trials, the subject rat was exposed to the same intruder. In the last trial (5^th^), a new intruder (2^nd^ intruder) was placed in the same acrylic chamber, which had been properly cleaned to remove the odor of the previous intruder, and the time the rat spent sniffing was re-quantified. The time spent sniffing in the social interactions was recorded and measured by a video camera (Sony, Madrid, Spain; #V88E) connected to a computer equipped with a computerized video-tracking system (EthoVision Pro, Noldus Information Technologies, Wageningen, The Netherlands). The observer was blinded to phenotype and treatment.

The duration of time the host rat spent exploring, by sniffing the intruder through the orifices, was used as a measure of social recognition. Social recognition was reflected in a reduction in the time spent sniffing between the 1^st^ and 4^th^ trials. Reaction to novelty was reflected in an increase in the time spent sniffing in the 5^th^ trial compared to the 4^th^ trial [40].

Hippocampal function was measured through the spatial reference memory task in two different mazes, the radial arm water maze (RAWM) and the Barnes maze, whereas prefrontal cortex function was measured through the spatial working memory task in the RAWM. The RAWM consists of four black fiberglass arms (80 cm x 12 cm each arm) in the shape of a cross. The pool, filled with water up to 30 cm at a temperature of 22 ± 2 °C, contained a cylindrical escape platform for the animals measuring 10 cm in diameter and 28 cm in height, of which 2 cm was submerged in water. The RAWM was in the center of a 16 m^2^ lit room (50 lx in the center of the maze), surrounded by panels on which several extra-maze visual clues with different colors and shapes were placed above four quadrants of the maze. One day prior to the spatial task, the experimental groups received a habituation session consisting of three trials with the platform, using different starting positions each time in a small square water tank (47 × 75 × 38 cm).

The spatial reference memory task in the RAWM was evaluated for five days (n/group = 6), with the escape platform maintained in a fixed position every day (arm D). Each daily session was composed of six trials. During the training period, the rat was placed in the center of the maze facing an arm determined by a pseudo-randomized sequence, and the rat was allowed to swim around the maze for 1 min to find the escape platform. If the rat failed to find the escape platform within 1 min, it was gently guided to the platform and remained there for 15 s. At the end of the session, a probe test was applied in which the escape platform was removed, and the rat was introduced into the maze for 2 min in order to check whether the animal remembered the position of the escape platform. Immediately after the probe test, the animals were subjected to an additional trial with the escape platform placed in its usual position to preclude any possible interference with the probe test. The time the rat spent to find where the escape platform used to be located (latency), distance travelled, speed, and percentage of correct choices were recorded and analyzed by EthoVision Pro (Noldus Information Technologies, Wageningen, The Netherlands).

To perform the spatial reference memory task in the Barnes Maze (122 cm diameter and 70 cm height), an escape box was placed under one of the holes on the maze and remained in the same spot during the course of the 5-day training session (n/group = 5). Visual cues of different colors and shapes were placed above four quadrants of the maze (1200 lx in the center of the maze). In the acclimation period, each rat was placed at the edge of the escape box. The rat was gently placed into the escape box if it had not entered it on its own within 1 min. During the training period, the rat was placed in the center of the maze and covered by an opaque plastic box. On each training trial, 11 of the 12 holes were blocked. The remaining hole provided access to the escape box, which was positioned on the underside of the maze. The ramp leading to the escape box was of the same color and texture as the doors blocking the holes, such that it was visually indistinguishable from the 11 other holes from the center of the maze. After 15 s, the box was removed, and the rat was allowed to walk around the maze for 3 min to find and enter the escape box. If the rat failed to enter the escape box within 3 min, it was gently guided to the box. At the end of the session, a probe test was applied in which the escape box was removed, and the rat was introduced into the maze for 2 min in order to check whether the animal remembered the position of the escape box. Immediately after the probe test, the animals were subjected to an additional trial, with the escape box placed in its usual position to preclude any possible interference with the probe test. Four trials were performed every day for five days. The time spent to find the target hole where the escape box used to be located (latency), distance travelled, and speed were recorded and analyzed by EthoVision Pro (Noldus Information Technologies, Wageningen, The Netherlands). The maze was wiped thoroughly with a 10% alcohol solution to remove any potential olfactory cues.

In contrast, prefrontal cortical function was evaluated through the spatial working memory test in the RAWM, for which training involved a paired sample task on five days of training. Each daily session consisted of two identical trials (sample and retention). In both trials, the escape platform was maintained in a fixed position every day, alternating across days in a pseudorandom order. Within the same session, the platform, which was not visible to the animals because it was hidden under water, was situated in one of the four arms. With the animals starting at the center of the maze facing an arm, each rat was allowed to explore the maze freely. If the rat did not find the escape platform within 60 s, it was gently picked up by the experimenter and placed on the platform. The rat remained in the escape platform for 15 s before it was returned to its home cage for 5 s (intertrial interval). Training ended when the group achieved the learning criteria, established as a statistically significantly lower retention latency compared to both the sample latency within one session and to the retention latency from the first session [41]. Latencies (s) to reach the platform were recorded.

### Assessment of brain metabolic activity: Cytochrome c oxidase

The cytochrome c oxidase (CCO) histochemistry (n/group = 9) protocol provides a measure of metabolic brain activity based on the method developed by Gonzalez-Lima and Cada [42], which consists of a modified version of the protocol described by Wong-Riley [43]. Ninety minutes after the last session of the spatial working memory task, the animals were decapitated. The whole brain hemisphere was then processed in 30 μm-thick coronal sections using a cryostat microtome (Leica, Wetzlar, Germany; #CM1900) and subsequently mounted on slides. In order to control staining variability across different baths, sets of brain tissue homogenate standards of known CCO activity from rat brains were cut at different thicknesses (10, 30, 50 and 70 μm) and included with each batch of slides [44]. Sections and standards were fixed with a 0.5% (v/v) glutaraldehyde (Merck, Darmstadt, Germany), 10% (w/v) sucrose (Sigma-Aldrich, St. Louis, MO, USA) in phosphate buffer (0.1 M, pH 7.6) solution. Next, the slides were rinsed three times in 10% (w/v) sucrose (Sigma-Aldrich, St. Louis, MO, USA) in phosphate buffer (0.1 M, pH 7.6) and immersed in a Tris (Sigma-Aldrich, St. Louis, MO, USA) buffer solution (0.05 M, pH 7.6) containing 0.5% (v/v) dimethylsulfoxide (Fisher Scientific, Hampton, VA, USA), 10% (w/v) sucrose (Sigma-Aldrich, St. Louis, MO, USA), and 275 mg/L cobalt chloride (Sigma-Aldrich, St. Louis, MO, USA). Once the slides had been rinsed in a phosphate buffer (0.1 M, pH 7.6), they were incubated in the dark at 37°C in a PBS solution (0.1 M, pH 7.6) containing 0.0075% (w/v) cytochrome c (Sigma-Aldrich, St. Louis, MO, USA), 0.002% (w/v) catalase (Alfa Aesar, Haverhill, NH, USA), 5% (w/v) sucrose (Sigma-Aldrich, St. Louis, MO, USA), 0.25% (v/v) dimethylsulfoxide (Fisher Scientific, Hampton, VA, USA), and 0.05% (w/v) diaminobenzidine tetrahydrochloride (Sigma-Aldrich, St. Louis, MO, USA) for 1 h. The reaction was stopped by fixing the tissue in 4% (v/v) buffered formalin (Sigma-Aldrich, St. Louis, MO, USA) with 10% (w/v) sucrose (Sigma-Aldrich, St. Louis, MO, USA). Finally, the slides were dehydrated, cleared with xylene, and coverslipped with Entellan (Merck, Darmstadt, Germany).

The CCO histochemical staining intensity was quantified by densitometric analysis using the computer-assisted image analysis workstation MCID (Interfocus Imaging, Linton, United Kingdom), consisting of a high precision illuminator, a digital camera, and a computer with specific image analysis software. The mean optical density (OD) of each region was measured by taking twelve readings which were then averaged to obtain one mean per region for each animal. These OD values were then converted to CCO activity units (μmol of cytochrome c oxidized/min/g tissue wet weight), determined by the enzymatic activity of the standards measured spectrophotometrically.

In all the animals, we measured the neuronal metabolic activity in selected brain regions that were anatomically defined according to Paxinos and Watson’s atlas [45]. The regions of interest and their distance from bregma were: the prefrontal cortex (+3.24 mm) (prelimbic (PrL), infralimbic (IL) and cingulate (Cg) cortex), the dorsal striatum (STD) (+1.56 mm), the ventral striatum (+1.56 mm) (accumbens core (AcbC) and shell (AcbSh)), the thalamus (-1.20 mm) (anterodorsal (ADT), anteroventral (AVT) and mediodorsal (MDT)), the amygdala (-2.28 mm) (central (CeA), basolateral (BLA) and lateral (LaA)), the dorsal hippocampus (-3.00 mm) (dentate gyrus (DG), CA1 and CA3 areas), the hypothalamic nuclei (-3.24 mm) (ventromedial (VMH) and dorsomedial nucleus (DMH)) and the mammillary bodies (-4.44 mm) (medial nucleus (mMM), the lateral part of the medial nucleus (lMM), the lateral mammillary nucleus (LM), and the supramammillary nucleus (SuM)).

### Monoamine determination

Monoaminergic activity was evaluated by assessing serotonin (5-HT), dopamine (DA), and noradrenaline (NA) levels in the prefrontal cortex, striatum, hippocampus, and cerebellum. Their respective metabolite levels, including 5-hydroxyindoleacetic acid (5-HIAA), 3,4-dihydroxyphenylacetic acid (DOPAC), and 3-methoxy-4-hydro-xyphenylglycol (MHPG) were also analyzed. Moreover, 5HIAA/5HT, DOPAC/DA, and MHPG/NA ratios were calculated (n/group = 7).

These results were obtained via high-performance liquid chromatography (HPLC), using an Agilent 1200 LC system (Agilent Technologies, Madrid, Spain) equipped with a vacuum degasser, quaternary pump, cooled autosampler, thermostatted column compartment, and fluorescence and variable wavelength detectors. The chromatographic separation was performed on a Poroshell 120 EC-C18 column (100 × 4.6 mm, 2.7 μm) protected by a cartridge guard column (Agilent Technologies, Madrid, Spain). The mobile phase consisted of 0.05% trifluoroacetic acid (solvent A) and acetonitrile (solvent B). The flow was maintained at a constant rate of 0.5 ml/min. The gradient elution program was as follows: from 0 to 8 min, 6% solvent B (v/v); from 8 to 15 min, 10% solvent B (v/v); from 15 to 22 min, 20% solvent B (v/v); and from 22 to 25 min, 2% solvent B (v/v). The column was maintained at 25 °C during the analysis, and samples were maintained at 4 °C in an autosampler unit. The effluent was monitored with the fluorescence detector at excitation wavelengths of 283 nm for DOPAC and 5-HIAA, 212 nm for NA, MHPG, and DA, and 229 nm for 5-HT. For all analyses, the emission wavelength was 320 nm. The total sample analysis time was 22 min. The mobile phase was prepared daily and filtered through a 0.22-μm Durapore filter (Millipore, Madrid, Spain). Prior to the sample preparation, the frozen tissues were weighed on AG204 analytical scales (Mettler Toledo, Columbus, OH, USA). The tissues were homogenized and deproteinized in a 60 μl homogenization solution (1% formic acid in acetonitrile) in a Bullet Blender homogenizer (Next Advance, New York, NY, USA; #BBY24 M Bullet Blender Storm). The aforementioned treatment denatured the protein molecules, making the protein levels virtually impossible to detect. The homogenates were immediately vortexed for 5 min (Scientific Industries, Bohemia, NY, USA; Vortex-Genie 2) and subsequently centrifuged for 15 min at 15,000 × g and 4 °C (Beckman Coulter, Madrid, Spain; Microfuge #22R Centrifuge). The supernatants were dried for 30 min with compressed air to concentrate the samples and subsequently reconstituted with 30 μl of 0.05% trifluoroacetic acid. Given that it is impossible to filter such small volumes, the samples were centrifuged for 15 min at 15,000 × g and 4 °C. Ultimately, 20 μl of each supernatant was injected into the HPLC system for analysis. Data processing was performed with the Agilent ChemStation software program (Agilent Technologies, Madrid, Spain); this program was used to quantify all compounds by comparing the areas under the peaks with the areas of the reference standards. All standards were purchased from Sigma-Aldrich (St. Louis, MO, USA) and dissolved in a stock 0.1 N hydrochloric acid solution. The calibration samples were prepared by adding appropriate amounts of standard working solutions to chromatographic grade water obtained from a Millipore water purification system (Millipore, Madrid, Spain).

### Statistical analysis

Physiological and neurobiological data were analyzed in the SigmaStat 3.2 program (Systat, San Jose, CA, USA) and expressed as means ± SEM. A two-way repeated-measures ANOVA was used to compare weight gain across weeks, and a Student’s *t*-test was used to compare the relative organ weight, biochemical determination, and portal pressure measurements. Locomotor activity, anxiety-like behavior, anhedonia, despair, percentage of social interaction, CO quantification, and monoamine determination were analyzed using a Student´s *t*-test for independent variables. Two-way repeated-measures ANOVAs were used to analyze the interactions on the short-term social memory test, permanence in the correct quadrant while performing the Barnes maze, percentage of time spent in the correct arm when performing RAWM, latencies in the spatial memory tasks, as well as sample and retention analyses of working memory and duration of short-term social memory data. Post-hoc multiple comparison analyses were performed, when possible, using the Bonferroni method. A Mann–Whitney’s *U* test for independent samples and a Friedman repeated-measures analysis of variance on ranks were applied when tests to assess normality or equal variance across group failed.

Data derived from microbiota were analyzed using the IBM SPSS Statistics Version 24.0 (IBM, Armonk, NY, USA) software. A Mann–Whitney’s *U* test was used to analyze the 16S data. Data derived from qPCR and SCFAs were normally distributed and were analyzed using a Student’s t-test. Principal component analyses (PCA) were performed by using the function “cluster::pam” in the R program package (v3.5.2).

The results were considered statistically significant if *p*<0.05.

## Results

### Portal pressure

The NASH group had significantly elevated portal pressure compared to NC (NC: 7.018 ± 0.638; NASH: 9.915 ± 0.153 mmHg; *U* = 34.000, *n*_1_ = 4, *n*_2_ = 6, *p* = 0.010).

### Organ relative weight

**Fig 1:**
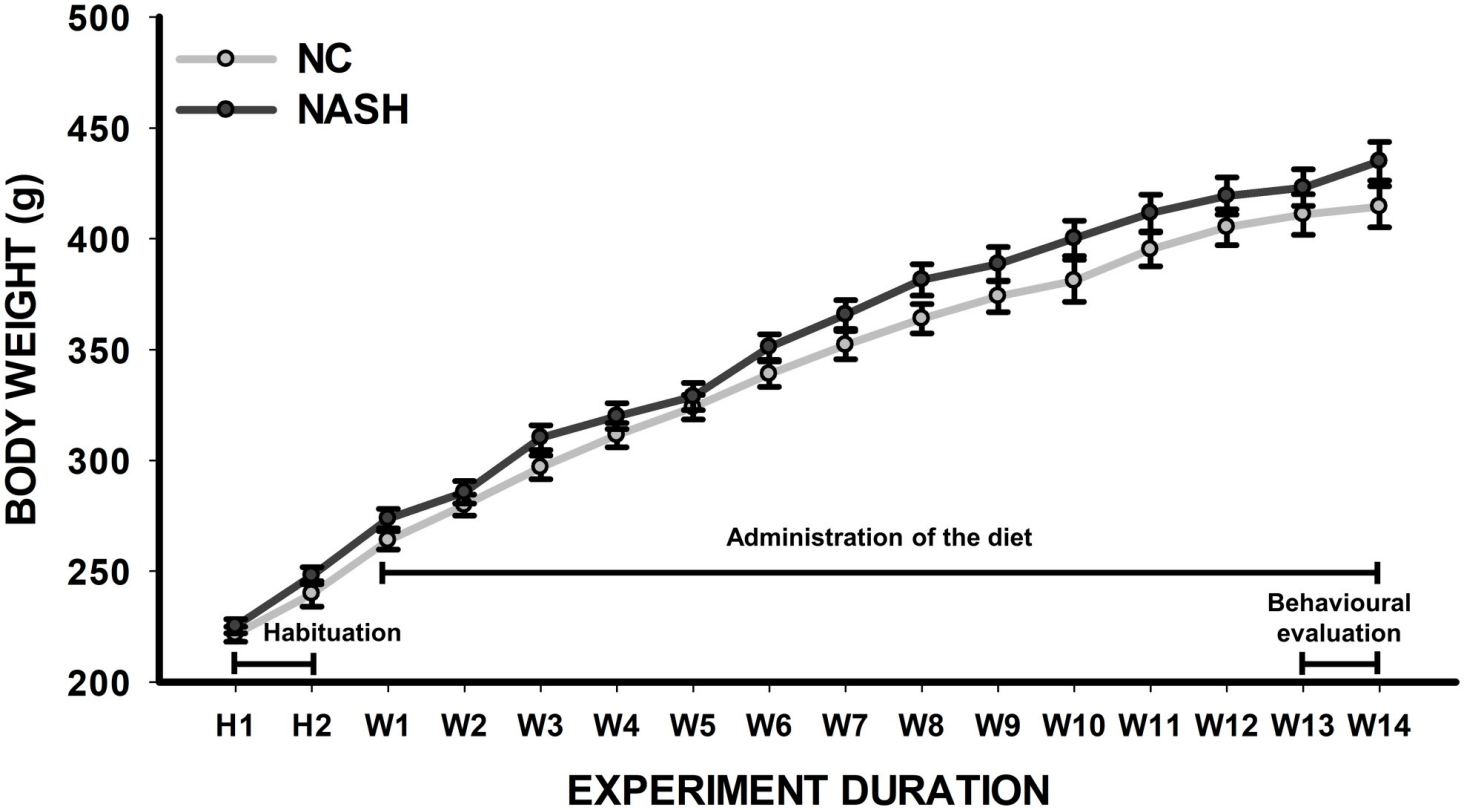
Body weight of Sprague-Dawley rats fed with a high-fat and high-cholesterol diet (NASH) and a standard diet (NC) throughout the 14 weeks of the study. A schematic representation of the experimental plan is also shown. No difference between groups was found (*p*≥0.05).

No statistically significant differences in the body weight between groups were found throughout the 14 weeks of the administration of the diet (*F*_1,479_ = 2.064, *p* = 0.162). As expected, significant differences between weeks were revealed (*F*_1,15_ = 862.136, *p*<0.001), showing normal weight gain according to age in both experimental groups (Fig 1). Nevertheless, differences were also revealed in the relative weight of some of the measured organs (organ weight/body weight). The NASH group had an increased relative weight of the liver (*t*_28_ = 11.041, *p*<0.001), spleen (U = 126.000, *n*_*1*_ = 15, *n*_*2*_ = 15, *p* = 0.002), and kidney (*t*_28_ = 3.479, *p* = 0.002). In contrast, compared to the NC group, the NASH group also had a decreased relative weight of the brain (*t*_28_ = 2.755, *p* = 0.010). No difference in the relative weight of the adrenal glands (*t*_28_ = 2.017, *p* = 0.053) was observed (Table 1).

**Table 1:** Body weight *(g)* and organs’ relative weight *(g/g)*.

	NC	NASH
**Body**	414.480±9.310	435.013±8.696
***Brain***	0.00453±0.000101	* 0.00407±0.000131
***Liver***	0.0320±0.000950	* 0.0462±0.000865
***Adrenal gland***	0.0000636±0.00000212	0.0000707±0.00000278
***Kidney***	0.00262±0.0000552	* 0.00288±0.0000497
***Spleen***	0.00179±0.0000328	* 0.00333±0.000150

Values represent mean ± SEM

*p<0.05

### Liver histological examination

**Fig 2:**
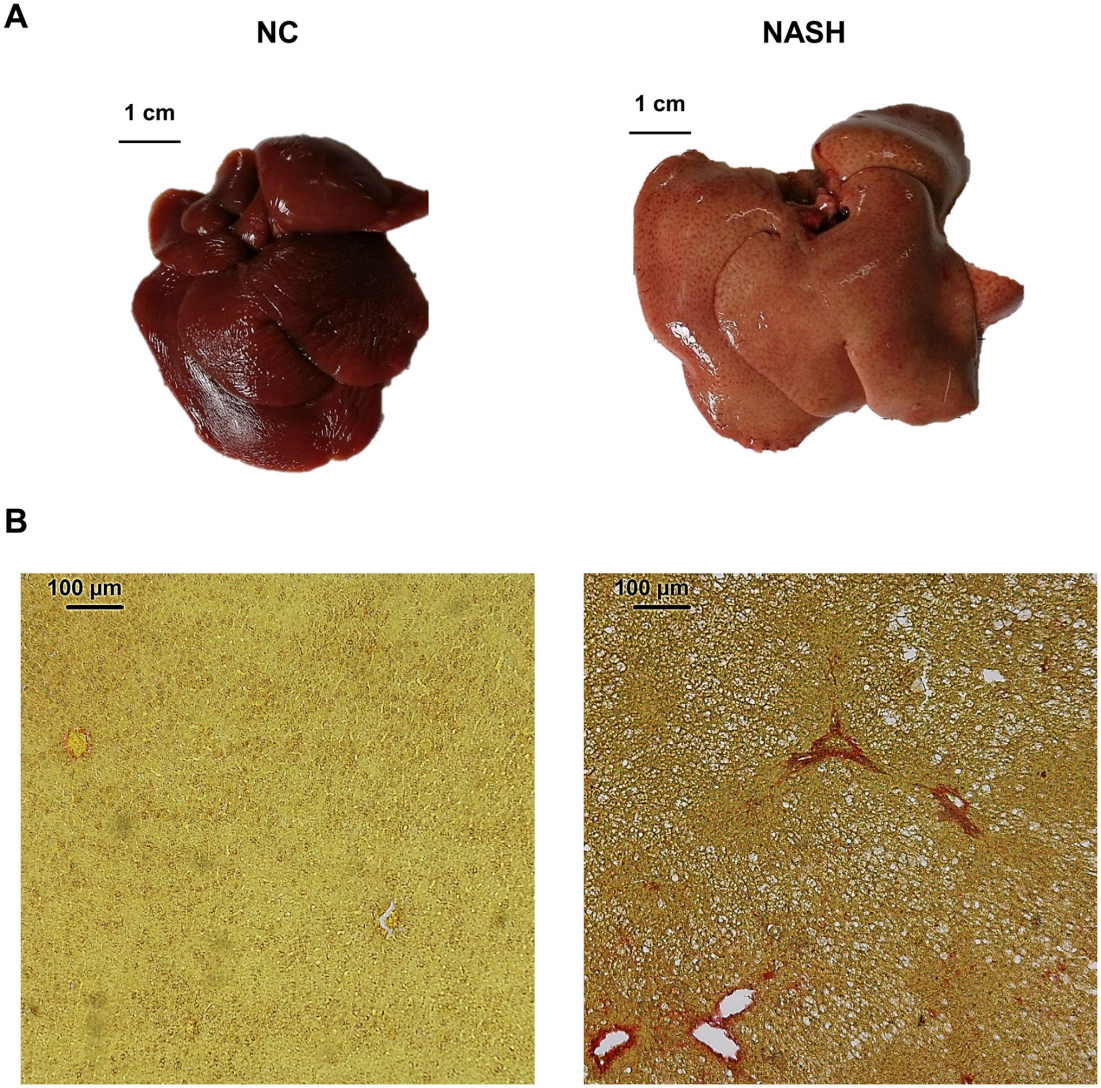
Liver examination. (**A**) Macroscopic view of NC and NASH livers. (**B**) Microscopic (10x) view of NC and NASH livers after being stained with picrosirius red. The NASH group presented with steatosis.

The NASH group presented with steatosis in their livers, reflected in an increased accumulation of lipids compared to the NC group (Fig 2).

### Biochemical plasma determination

Plasma biochemistry concentrations were assessed in both groups (Table 2). Compared to the NC group, the NASH group showed a significant increase in AST (*t*_21_ = 2.316, *p* = 0.031), ALT (*U* = 72.000, *n*_*1*_ = 11, *n*_*2*_ = 12, *p*<0.001), total protein (*t*_13_ = 4.624, *p*<0.001), glucose (*t*_9_ = 3.874, *p* = 0.004), total cholesterol (*U* = 16.000, *n*_*1*_ = 5, *n*_*2*_ = 7, *p* = 0.005), and LDL cholesterol (*t*_11_ = 5.686, *p*<0.001), as well as ammonia (*t*_14_ = 2.188, *p* = 0.046). Furthermore, the NASH group also had higher levels of creatinine (*t*13 = 16.757, *p*<0.001) and HDL cholesterol (*t*9 = 2.417, *p* = 0.039). Changes in albumin (*U* = 93.000, *n*_*1*_ = 10, *n*_*2*_ = 12, *p* = 0.156), urine (*t*13 = 0.0415, p = 0.968), bilirubin (*U* = 151.500, *n1* = 11, *n2* = 14, *p* = 0.661), and triglycerides (*U* = 93.000, *n1* = 9, *n2* = 11, *p* = 0.939) were not statistically significant.

**Table 2:** Biochemical values in plasma.

	NC	NASH
**Albumin** *g/L*	34.177±0.508	35.856±0.770
**NH**_**3**_ *U*	394.625±41.220	* 557.000±61.703
**AST** *U/L*	110.136±21.707	* 169.792±14.592
**ALT** *U/L*	45.145±5.069	* 124.517±16.466
**Total protein** *mg/dL*	50.840±1.123	* 60.260±1.821
**Glucose** *U*	119.000±6.380	* 144.667±2.963
**Creatinine** *μM/L*	86.617±3.855	* 30.533±1.044
**Urine** *μM/L*	6.061±0.277	6.080±0.331
**Bilirubin** *U*	0.714±0.172	0.509±0.0858
**Cholesterol** *U*	92.400±11.885	* 160.714±10.497
**HDL** *mg/dL*	80.750±19.964	* 43.429±4.029
**LDL** *mg/dL*	23.000±2.828	* 82.000±7.888
**Triglycerides** *mg/dL*	0.305±0.0435	0.323±0.0739

Values represent mean ± SEM

*p<0.05

### Microbiota exploration

#### Analysis of fecal microbial groups by 16S rRNA gene profiling

**Fig 3:**
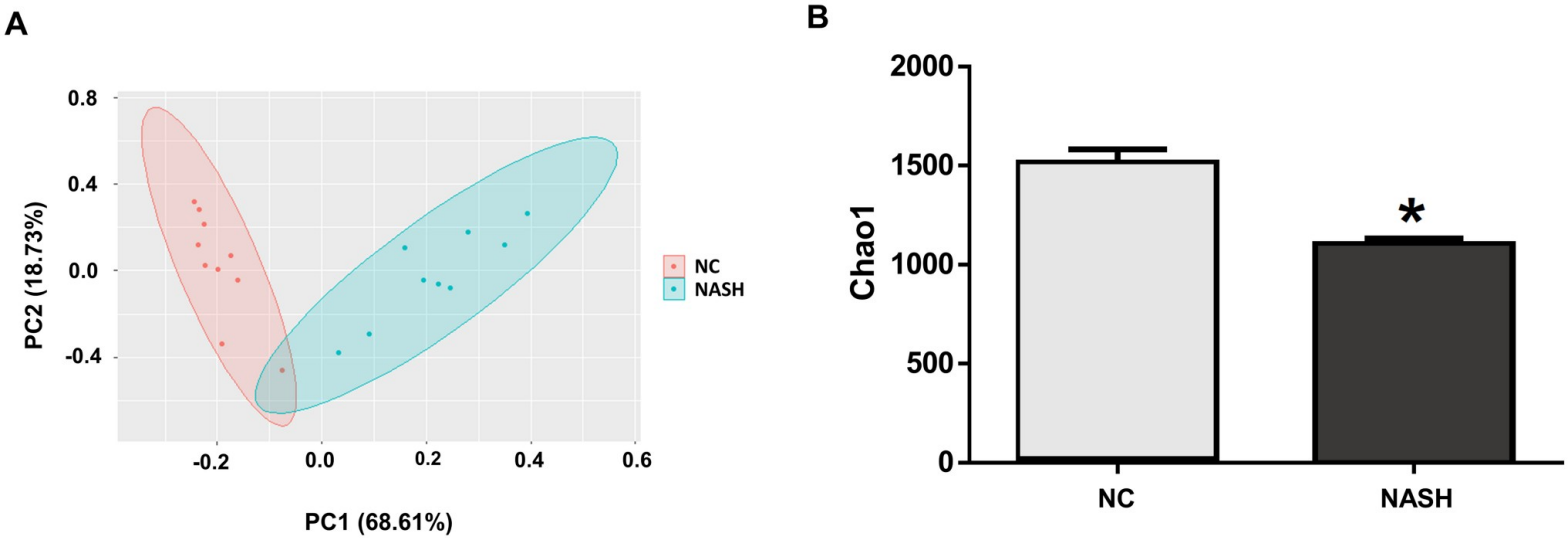
Microbial composition. (**A**) PCA obtained through cluster analysis of the NC (control group) and NASH group samples. (**B**) Alpha diversity estimated by the Chao1 diversity index for feces samples from the NC and NASH groups (*p<0.05).

**Fig 4:**
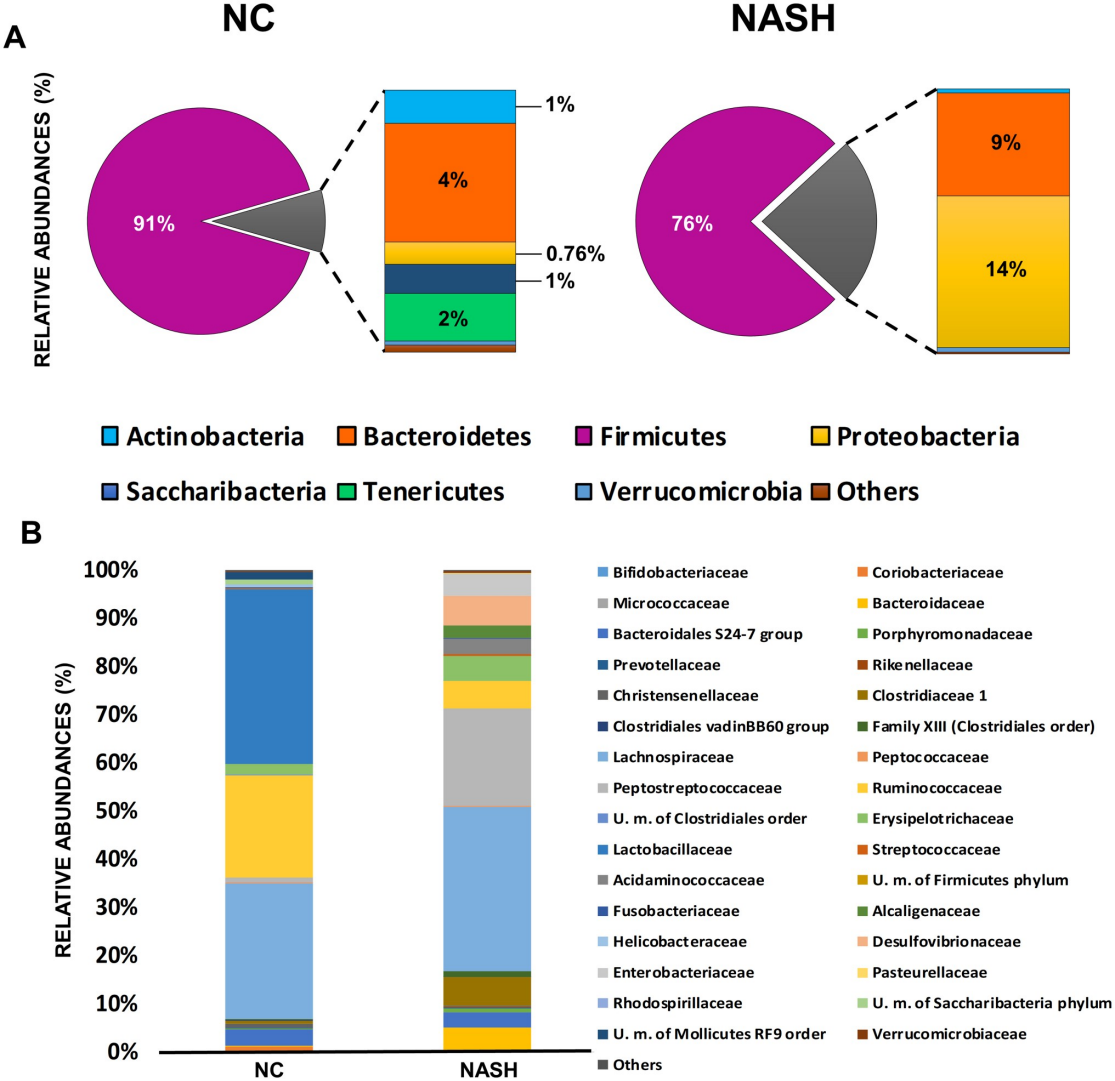
Microbiota population. Relative abundance (%) of bacteria in fecal samples from NC and NASH groups at (A) phylum and (B) family level.

MiSeq sequencing of the V3-V4 region of the 16S rRNA gene from fecal samples yielded an average of ~65,000 filtered partial sequences per sample, of a length of ~178 bp. The analyses of the 16S rRNA gene profiling of fecal samples showed large differences in the intestinal microbiota composition between the two groups, as evidenced by the clear separation seen in the principal components analysis (PCA) (Fig 3A). The evaluation of the Chao 1 richness estimator revealed significantly (*p* = 0.004) lower bacterial richness in the NASH group (Fig 3B). Taxonomic shifts were also assessed. At the phylum level, the murine fecal microbiota was dominated by Firmicutes (Fig 4A) in both groups. However, a significantly (*p* = 0.000) lower proportion of this phylum was observed in the NASH group, in which a significantly increased relative proportion of Proteobacteria (*p* = 0.000) and Bacteroidetes (*p* = 0.003) was observed. Analysis of the data at the family level confirmed these differences (Fig 4B). The NASH and NC groups also had differences in the relative abundance of families belonging to the Firmicutes phylum. The NC group harbored significantly (*p* = 0.000) higher proportions of *Ruminococcaceae* and *Lactobacillaceae*, whereas the NASH group had higher proportions (*p* = 0.000) of *Peptostreptococcaceae* and *Clostridiaceae* 1. In addition, in the NASH group, a significant (*p* = 0.000) increase in *Enterobacteriaceae*, *Desulfovibrionaceae* (both families belonging to the Proteobacteria phylum), and *Bacteroidaceae* was observed. Consistent with these results, differences were observed in the relative abundance of different microbial genera between the two groups (S1 Table). The NC group was dominated by and showed significantly (*p* = 0.000) higher proportions of *Lactobacillus* compared to the NASH group, in which higher (*p* = 0.000) proportions of *Bacteroides*, *Blautia*, *Romboustia*, *Bilophila*, or *Escheria-Shigella* were found.

#### Analysis of fecal microbial groups by quantitative PCR

Absolute levels of selected microbial groups were determined by qPCR (Table 3), which made it possible to confirm the observations from the 16S profiling. The NASH group showed significantly (*p* = 0.000) lower counts of total bacteria, *Lactobacillus*-group, *Clostridium* cluster IV, and *Bifidobacterium*, and significantly (*p* = 0.000) higher counts of *Enterobacteriaceae* bacteria. However, the increase observed in *Bacteroidaceae* levels by 16S analysis was not confirmed by qPCR. This inconsistency may be due to the specificity of the primers used for the *Bacteroides* since qPCR used for the group determination does not amplify all the family members.

**Table 3:** Concentration of intestinal microbial groups and levels of SCFAs.

**Microbial group** *(Log CFU/g)*	**NC**	**NASH**	***p-value***
***Akkermansia***	5.72±0.84	5.97±0.61	0.473
***Bacteroides* group**	9.44±0.68	8.72±0.43	* 0.014
***Bifidobacterium***	8.48±0.13	6.35±0.51	* 0.000
***Clostridium* Cluster IV**	7.88±0.27	6.99±0.50	* 0.000
***Clostridium* Cluster XIVa**	8.22±0.30	8.5±0.37	0.088
***Enterobacteriaceae***	5.59±0.48	8.29±0.78	* 0.000
***Lactobacillus* group**	9.21±0.20	5.45±0.22	* 0.000
**Total Bacteria**	10.72±0.30	9.78±0.41	* 0.000
**SCFA** *(mM)*	**NC**	**NASH**	***p-value***
**Acetate**	44.37±13.08	18.1±3.74	* 0.000
**Propionate**	8.29±2.06	5.68±2.15	* 0.015
**Butyrate**	13.36±7.18	2.34±0.67	* 0.001
**Total main SCFAs**	66.03±20.54	28.38±10.26	* 0.000

Values represent mean ± standard deviation

*p<0.05

#### Determination of SCFAs in feces

The levels of the main SCFAs derived from bacterial metabolism differed between the NC and NASH groups (Table 3). The latter group showed significantly lower concentrations of acetate (*p* = 0.000), propionate (*p* = 0.015), and butyrate (*p* = 0.001), as well as total SCFAs (*p* = 0.000). These results indicate a decrease in microbiota activity and metabolism.

### Behavioral evaluation

#### Test for assessment of locomotor activity: Rotarod-accelerod test

**Fig 5:**
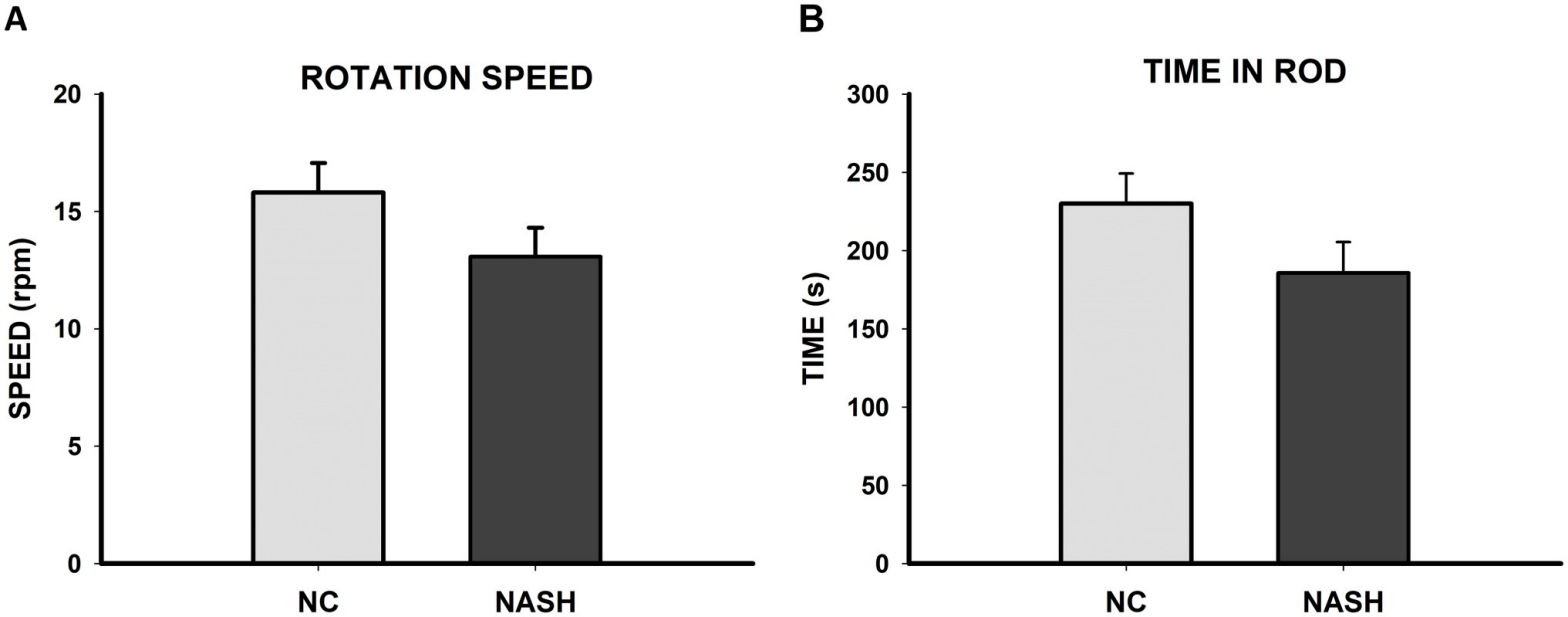
Locomotor activity. Bar charts (mean±SEM) represent (**A**) maximum rotation speed and (**B**) time spent on the apparatus. There were no statistically significant differences between groups and the NASH group did not show impaired locomotor activity.

Results show that were no statistically significant differences between groups in the rpm reached by the rotarod (*U* = 269.000, *n*_*1*_ = 15, *n*_*2*_ = 15, *p* = 0.127) (Fig 5A) or in the time spent on the rod (*t*_28_ = 1.611, *p* = 0.118) (Fig 5B), thereby indicating that the NASH group’s locomotor activity was not affected.

#### Tests for assessment of anxiety- and depressive-like behavior

**Fig 6:**
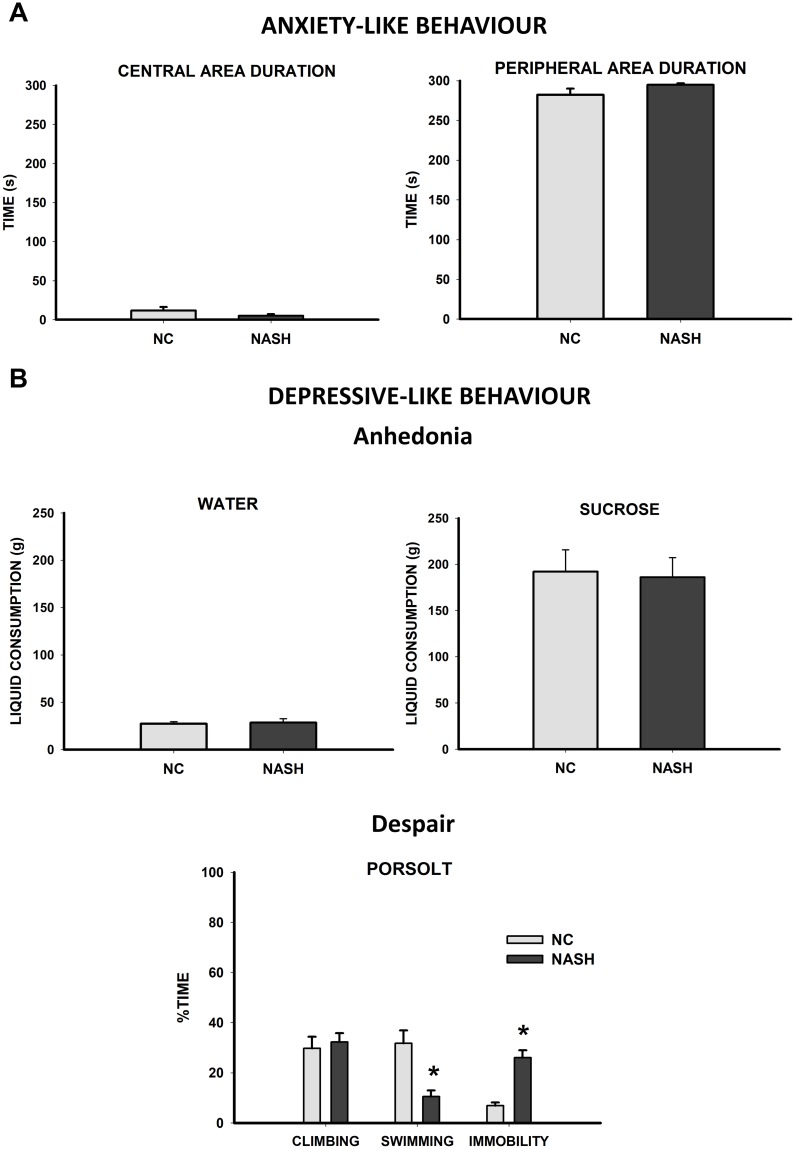
Assessment of anxiety- and depressive-like behavior. (**A**) **Anxiety-like behavior** was evaluated in the open field exploratory test. Bar charts (mean±SEM) represent time spent by the experimental groups in the central and peripheral areas. There were no statistically significant differences between groups and the NASH group did not show anxiety-like behavior. (**B**) **Depressive-like behavior** was evaluated by assessing anhedonia and despair. Anhedonia was measured with the sucrose preference test. Bar charts (mean±SEM) represent the 24 h consumption of water and sucrose. The NASH group did not show signs of anhedonia. Despair was assessed by the forced swimming test. Bar charts (mean±SEM) represent the percentage of time spent climbing, swimming, and in immobility in the NC and NASH groups. The NASH group spent significantly less time swimming and more time immobile than the NC group (*p<0.05), which is associated with depressive-like behavior.

In the evaluation of anxiety-like behavior in the open field, results revealed no statistically significant differences between groups in the time spent either in the center of the open field (*U* = 75.000, *n*_*1*_ = 15, *n*_*2*_ = 15, *p* = 0.124) or in the periphery (*U* = 194.000, *n*_*1*_ = 15, *n*_*2*_ = 15, *p* = 0.114). Therefore, no anxiogenic effects were found in the NASH group (Fig 6A).

The depressive-like behavior was evaluated through two different tests. Anhedonia was studied in the sucrose preference test, in which no differences were found in water (*U* = 256.000, *n*_*1*_ = 15, *n*_*2*_ = 15, *p* = 0.339) or sucrose consumption (*t*_28_ = 0.181, *p* = 0.857) between the groups (Fig 6B), meaning that the NASH group was not in an anhedonic state. In contrast, despair was evaluated by the forced swimming test (Porsolt) in which the NASH group spent less time swimming (*U* = 312.000, *n*_*1*_ = 15, *n*_*2*_ = 15, *p*<0.001) and more time immobile (*U* = 132.000, *n*_*1*_ = 15, *n*_*2*_ = 15, *p*<0.001) than the NC group, but did not differ in terms of climbing time (*U* = 210.000, *n*_*1*_ = 15, *n*_*2*_ = 15, *p* = 0.360) (Fig 6C). Thus, during this task, the NASH group showed signs of despair, a component of depression.

#### Tests for assessment of cognitive function

**Fig 7:**
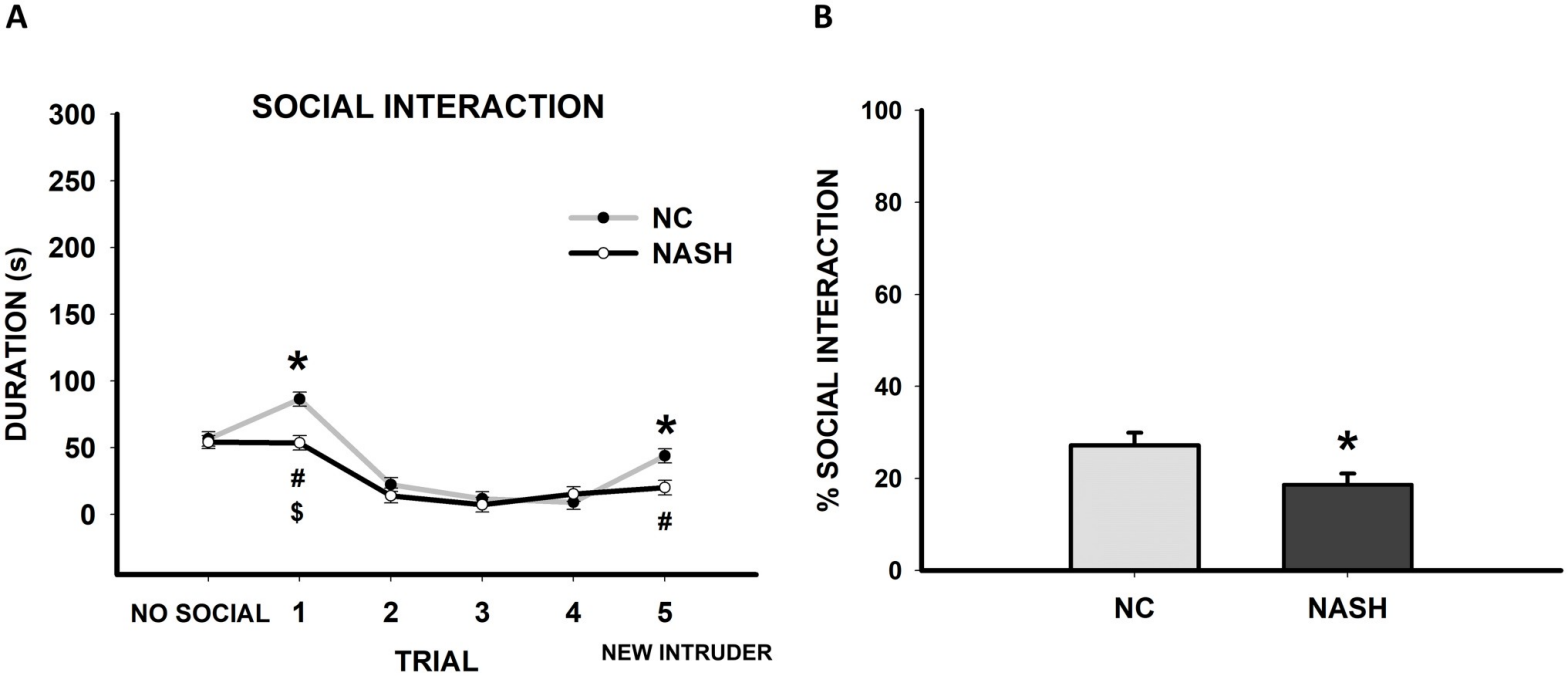
Cognitive assessment: Short-term social memory. (**A**) The scatterplot (mean±SEM) shows the duration of social interaction between groups on the non-social trial, in each of four successive trials and in the fifth trial. The NASH group spent significantly less time interacting with the social stimulus than the NC group in trials 1 and 5 (*p<0.05). There was a statistically significant reduction in the duration of the interaction between trials 3, 2, and 1 in the NC group (#*p*<0.05) and between trials 1 and 5 in the NASH group ($*p*<0.05). (**B**) Bar charts (mean±SEM) represent the percentage of time spent exploring the social stimulus. The NASH group showed significantly less interaction time than the NC group (*p<0.05). In summary, the NASH group showed impaired social behavior.

In the short-term social memory test, no differences were found between the experimental groups (*t*_22_ = 0.320, *p* = 0.752) during the non-social trial. However, in the social interaction experiment, differences were found between the experimental groups (*F*_1,124_ = 10.074, *p* = 0.004). In the first trial, the NC group showed extensive exploration of the intruder, whereas the duration of the interaction in the NASH group was significantly lower (*p*<0.001). This response decreased across trials with subsequent exposures in both the NC and NASH (*F*_4,124_ = 42.785, *p*<0.01) groups. When an unfamiliar rat (5^th^ trial) was introduced, the NC group again increased the time spent exploring, which was significantly higher than the time the NASH group rats spent interacting (*p* = 0.003). Moreover, interactions between group and trial were found (*F*_4,124_ = 4.211, *p* = 0.004). Specifically, the NC group showed differences between trials 1 and 5 and the rest of the trials (*p*<0.001), whereas the NASH group only showed differences between trials 1 and 5 (*p*<0.001) (Fig 7A). On analyzing the total social exploration, the NASH group spent statistically significantly less time exploring the intruder than the NC group (*t*_22_ = 2.285, *p* = 0.032) (Fig 7B). Thus, the NASH group can be considered to have demonstrated impaired short-term social memory.

**Fig 8:**
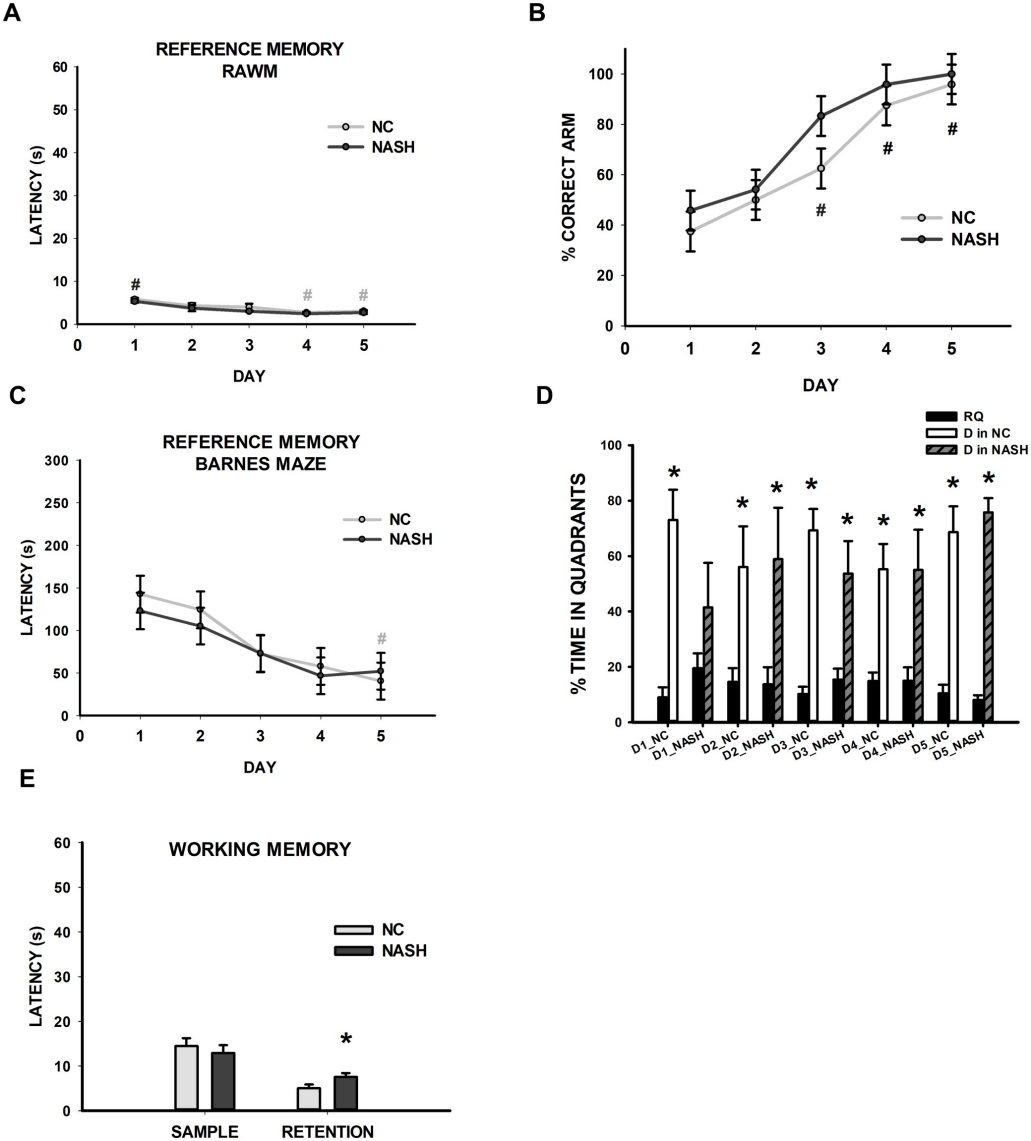
Cognitive assessment: Spatial reference and working memory. (**A**-**B**) **Spatial reference memory test to assess hippocampal function in the RAWM**: (**A**) The scatterplot (mean±SEM) shows escape latencies on training days. There was a statistically significant decrease in the duration of the interaction between days 4, 5, and 1 in the NC group and between day 1 and the other days in the NASH group (#*p*<0.05). (**B**) The scatterplot (mean±SEM) shows the percentage of time spent in the correct arm across the training days. The NASH group did not show impaired spatial reference memory, which is a hippocampal-dependent ability. (**C**-**D**) **Spatial reference memory test to assess hippocampal function in the Barnes Maze**: (**C**) The scatterplot (mean±SEM) shows escape latencies on training days. There was a statistically significant decrease in the duration of the interaction between days 5 and 1 in the NC group (#*p*<0.05). (**D**) Bar charts (mean±SEM) represent the amount of time spent in the rest of the quadrants (RQ: A, B, and C) compared to quadrant D, where the platform was located, in both the NC and NASH groups across training days. Both groups spent more time in arm D than in the rest of the arms over the course of the five days of training (*p<0.05). (**E**) **Spatial working memory test to assess prefrontal function**. Bar charts (mean±SEM) represent sample and retention average latencies. Whereas sample latencies were not statistically different, NASH animals showed a significantly higher retention latency than the NC group (*p<0.05). This group showed impaired spatial working memory, which is a prefrontal cortex-dependent function.

During the performance of the spatial reference memory test in the RAWM, the latency to reach the platform did not differ between groups (*F*_1,59_ = 2.907, *p* = 0.119), nor did the interactions (*F*_4,59_ = 0.221, *p* = 0.925). However, differences between days were found (*F*_4,59_ = 13.853, *p*<0.001); the NC group showed a decrease in latencies on days 4 and 5 (*p*≤0.05), whereas the NASH group showed significantly decreased latencies every day compared to day 1 (*p*<0.05) (Fig 8A). The distance traveled differed across days (*F*_4,59_ = 11.861, *p*<0.001), and the post-hoc analysis revealed significant differences between the first two days compared to the other days (*p*<0.05). There were no significant differences between groups on this measure (*F*_1,59_ = 2.503, *p* = 0.145), and no day×group interaction was found (*F*_4,59_ = 0.139, *p* = 0.967). The speed changed according to the days (*F*_4,59_ = 6.061, *p*<0.001), and the post-hoc analysis revealed significant differences between the first day and days 4 and 5 (*p*<0.020), as well as between day 2 and days 4 and 5 (*p*<0.05). There were no significant differences between groups on this measure (*F*_1,59_ = 0.880, *p* = 0.370) and no day×group interaction was found (*F*_4,59_ = 0.167, *p* = 0.954).

With regards to the reference memory percentages in the correct arm, these varied as the days went by (*F*_4,59_ = 19.039, *p*<0.001). There were no differences between groups on this measure (*F*_1,59_ = 2.788, *p* = 0.126), and the day×group interaction was not significant (*F*_4,59_ = 0.376, *p* = 0.824) (Fig 8B). Therefore, both experimental groups showed preserved spatial reference memory.

Spatial reference memory was also evaluated using a different paradigm, the Barnes maze, in both experimental groups. To this end, a hippocampal-dependent task was used in which the animals were required to locate an escape box that consistently remained in the same position. Our results revealed no differences in the latencies (*F*_1,49_ = 0.0548, *p* = 0.821) between the NC and NASH groups. However, differences in training days were found in the NC group between days 1 and 5 (*F*_4,49_ = 6.622, *p*<0.001), indicating that they were performing better as the training progressed (Fig 8C). To support the absence of locomotor problems, we analyzed distance and speed in both experimental groups while performing the spatial reference memory task. The distance traveled did not differ between the experimental groups (*F*_1,59_ = 0.08, *p* = 0.789), whereas differences in this measure were found across days (*F*_4,59_ = 6.102, *p* = 0.002), showing that the NC group travelled shorter distances from day 1 (*p*<0.005), whereas the NASH group started to improve from day 2 (*p* = 0.046). The day×group interaction was not significant (*F*_4,59_ = 2.257, *p* = 0.099). The speed differed as the days went by (*F*_4,59_ = 6.607, *p* = 0.001), and the post-hoc analysis showed significant differences in the NASH group between the first day and days 2, 3, and 4 (*p*<0.03). There were no significant differences between groups on this measure (*F*_1,59_ = 0.844, *p* = 0.400). The day×group interaction was not significant (*F*_4,59_ = 2.194, *p* = 0.107).

Further, a probe test was performed to determine whether the animals learned the exact position of the escape box. If the animals were able to remember the position, they would remain longer in the quadrant hosting the escape box (quadrant D); otherwise, they would spend more time in the rest of the quadrants (RQ). Both groups stayed longer in arm D, where the escape box was previously located, than in the rest of the arms (RQ) (NC: D1 (*F*_1,9_ = 30.820, *p*<0.001), D2 (*F*_1,9_ = 7.163, *p* = 0.028), D3 (*F*_1,9_ = 52.586), *p*<0.001), D4 (*F*_1,9_ = 17.464, *p* = 0.003), D5 (*F*_1,9_ = 35.113, *p*<0.001); NASH: D1 (*F*_1,9_ = 1.679, *p* = 0.231), D2 (*H*_1_ = 6.818, *p* = 0.008), D3 (*H*_1_ = 6.818, *p* = 0.008), D4 (*F*_1,9_ = 6.879, *p* = 0.031), D5 (*F*_1,9_ = 155.416, *p*<0.001; Fig 8D).

Prefrontal cortical function was evaluated via the spatial working memory test in the RAWM. The two-way repeated-measures ANOVA revealed no differences in the sample latencies between groups (*F*_1,89_ = 0.397, *p* = 0.538) across days (*F*_4,89_ = 2.351, *p* = 0.063). The NASH group showed a statistically significant higher retention latency than the NC group (*F*_1,89_ = 4.596, *p* = 0.048) (Fig 8E), meaning that the NASH group had impaired spatial working memory.

### Assessment of brain metabolic activity: Cytochrome c oxidase

The NASH group showed significantly lower values of CCO activity, indicating lower metabolic activity than the NC group, in IL (*t*_15_ = 2.568, *p* = 0.021), Cg (*t*_15_ = 2.814, *p* = 0.013), MDT (*t*_16_ = 5.352, *p*<0.001), CA1 (*t*_16_ = 2.927, *p* = 0.010), and LM (*t*_14_ = 4.077, *p* = 0.001).

However, no statistically significant differences were found in the CCO values between groups in PrL (*t*_15_ = 1.160, *p* = 0.264), STD (*t*_16_ = 1.093, *p* = 0.291), ADT (*t*_16_ = 0.157, *p* = 0.877), AVT (*t*_16_ = 1.473, *p* = 0.160), BLA (*U* = 81.000, *n*_*1*_ = 8, *n*_*2*_ = 9 *p* = 0.413), CeA (*t*_9_ = 3.110, *p* = 0.013), LaA (*U* = 86.000, *n*_*1*_ = 8, *n*_*2*_ = 9, *p* = 0.194), DG (*U* = 75.000, *n*_*1*_ = 9, *n*_*2*_ = 9 *p* = 0.377), CA3 (*t*_16_ = 1.256, *p* = 0.227), VMH (*t*_16_ = 0.535, *p* = 0.600), DMH (*t*_16_ = 0.0836, *p* = 0.934), mMM (*t*_14_ = 1.523, *p* = 0.150), lMM (*t*_14_ = 2.001, *p* = 0.065), or SuM (*t*_14_ = 0.841, *p* = 0.414) (Table 4).

**Table 4:** Metabolic brain activity.

Brain region	NC	NASH
**PrL**	28.380±1.030	26.735±0.975
**IL**	29.028±0.902	* 25.865±0.840
**Cg**	28.872±0.945	* 25.357±0.825
**STD**	26.193±0.546	26.931±0.397
**ADT**	36.116±0.606	36.343±1.314
**AVT**	31.348±0.774	29.661±0.845
**MDT**	26.949±0.818	* 21.739±0.528
**CeA**	27.425±0.868	25.483±0.518
**BLA**	29.171±0.723	28.103±1.014
**LaA**	24.412±0.511	22.224±1.106
**DG**	33.018±0.526	34.928±1.620
**CA1**	23.540±0.644	* 21.071±0.544
**CA3**	23.023±0.827	21.858±0.419
**VMH**	33.195 ± 1.269	32.342 ± 0.967
**DMH**	23.522 ± 1.121	23.405 ± 0.848
**mMM**	25.410±1.218	22.969±1.049
**lMM**	24.550±0.786	21.975±0.953
**LM**	23.963±0.861	* 19.724±0.629
**SuM**	23.982±1.144	22.916±0.683

*CCO activity units* represented as mean ± SEM

*p<0.05

### Monoamine determination

Neurotransmitters in both groups were analyzed in the prefrontal cortex, striatum hippocampus, and cerebellum. The NASH group presented lower levels of DA in the prefrontal cortex (*U* = 72.000, *n*_*1*_ = 7, *n*_*2*_ = 7, *p* = 0.011), an increased DOPAC/DA ratio in the cerebellum (*t*_12_ = 2.235, *p* = 0.045), and decreased NA (*U* = 69.000, *n*_*1*_ = 7, *n*_*2*_ = 7, *p* = 0.038) in the striatum compared to the NC group. There were no statistically significant differences in 5HT, 5HIAA, 5HIAA/5HT, DOPAC, MHPG, or MHPG/NA in the prefrontal cortex, striatum, hippocampus, or cerebellum (Table 5).

**Table 5:** Brain contents of monoamine neurotransmitters and their metabolites.

Monoamine *(nmol/g tissue)*		Prefrontal cortex	Striatum	Hippocampus	Cerebellum
**5 HT**	***NC***	0.02732±0.00975	0.02521±0.00570	0.00984±0.00390	0.01316±0.00279
	***NASH***	0.01056±0.00196	0.02775±0.00676	0.01397±0.00174	0.02296±0.00371
**5HIAA**	***NC***	0.05947±0.02480	0.03689±0.00283	0.02830±0.00309	0.01230±0.00135
	***NASH***	0.02137±0.00502	0.03083±0.00220	0.02223±0.00128	0.01865±0.00478
**DA**	***NC***	0.01695±0.00588	0.03946±0.01363	0.01333±0.00503	0.01400±0.00268
	***NASH***	*0.00306±0.00054	0.07776±0.01455	0.00500±0.00109	0.01204±0.00120
**DOPAC**	***NC***	0.37606±0.14598	0.04371±0.0123	0.03830±0.00869	0.08690±0.01475
	***NASH***	0.03816±0.00168	0.03659±0.00641	0.03200±0.00267	0.17354±0.04088
**NA**	***NC***	0.00847±0.00166	0.01174±0.00321	0.01149±0.00242	0.01027±0.00148
	***NASH***	0.01024±0.00151	*0.00399±0.00053	0.01099±0.00170	0.00960±0.00179
**MHPG**	***NC***	0.06983±0.03539	0.05934±0.02346	0.05180±0.00953	0.12201±0.03389
	***NASH***	0.02919±0.00606	0.02999±0.00521	0.04146±0.00975	0.10650±0.02800
**5HIAA/5HT**	***NC***	4.64425±3.36298	2.03764±0.56651	6.17124±1.72689	1.18908±0.23711
	***NASH***	2.1076±0.48026	1.63749±0.38179	1.74190±0.25556	0.82319±0.11745
**DOPAC/DA**	***NC***	19.27120±4.35385	2.86692±1.48622	4.46313±0.81159	7.37821±1.62266
	***NASH***	14.22486±1.81051	0.72799±0.25240	10.02642±3.22583	*13.96451±2.45926
**MHPG/NA**	***NC***	6.74773±2.10508	6.90871±1.78199	6.17729±1.95395	11.19352±1.78506
	***NASH***	3.39489±1.00960	7.64958±1.21543	4.33613±1.11574	12.15172±3.12631

Values represent mean ± SEM

*p<0.05

## Discussion

The purpose of this study was to investigate the consequences of HFHC consumption in an animal model that reflects some of the pathophysiological processes characteristic of human NASH pathology. We measured a set of clinical criteria characterizing the NASH condition. We investigated the role of gut microbiota composition and SCFAs in NASH-induced behavioral deficits. Finally, we also extended our analysis to evaluate the effects of this diet on brain metabolic dysfunction and its impact on neurotransmission and behavioral performance.

Our results showed that the NASH group had increased portal pressure, which was previously found to result from steatosis, intrahepatic resistance, and increased splanchnic blood flow [46]. Animals in both experimental groups had increased weights according to their age, but there were no differences in body weight between the two groups, indicating that these animals did not develop obesity [47]. However, the NASH group had significantly heavier livers than the NC group and presented with steatosis similar to that of human NASH [48]. Animals in the NASH group also had relatively heavier kidneys, which could be related to the increased prevalence of chronic kidney disease among affected individuals [49], as well as to splenomegaly, which probably resulted from portal hypertension [6].

The NASH group met the biochemical criteria characteristic of the disease because they had elevated levels of AST, ALT, and glucose [50], alongside reduced levels of liver function-associated creatinine [51]. Total cholesterol and LDL cholesterol levels were increased, whereas HDL cholesterol levels were reduced, suggestion that the administration of this diet not only causes higher cholesterol levels, but also shifts the balance towards the more harmful cholesterol types.

The NASH group was also associated with higher levels of ammonia. Ammonia is a well-known neurotoxin produced from urea by the action of bacterial ureases in the intestinal tract. Gut-derived ammonia is taken up by the liver and consumed in the urea cycle. It has been recently reported that steatosis in rats and humans is associated with reversible changes in urea cycle enzymes and impairment of urea synthesis [7]. In cirrhosis, studies have shown that, in addition to its direct neurotoxic injury, ammonia is also able to impair the intracerebral synthesis of serotonin and dopamine and produce abnormal neurotransmitters such as octopamine [52]. Additionally, hyperammonemia has been associated with neuroinflammation, which contributes to cognitive impairment [53,54]. These findings suggest that hyperammonemia could also be a contributing factor to the behavioral deficits in NASH.

Extensive literature has described the brain’s sensitivity to diet-induced obesity [55,56] and a growing number of studies have linked gut microbiota to central nervous system health and behavior [57–59]. However, this study’s results provide the very first evidence that high-fat high-cholesterol diet-induced changes to the gut microbiome are sufficient to disrupt brain metabolism and function in the absence of obesity. The NASH group showed intestinal microbiota dysbiosis in which levels of total bacteria, as well as β- and α-diversity, were reduced. Moreover, our results showed that the NASH model is associated with reduced levels of Firmicutes, especially members of the *Lactobacillaceae* family, and increased levels of Bacteroidetes and Proteobacteria. These results differ from those of studies on high-fat diet models that found a reduction in Bacteroidetes and an increase in Firmicutes [60,61]. However, both results support the hypothesis that the balance between these two phyla might reflect the balance between unhealthy and healthy microbiota [62]. Dysbiotic gut microbiota have been pointed out as a major source of ammonia and a cause of neuroinflammation in cirrhosis [63,64]. Similarly, gut dysbiosis has been linked to an alteration of inflammatory pathways [65], which has been observed in NASH [8] and could result from the rise in metabolic endotoxemia [66]. It has been also reported that members of the *Lactobacillaceae* family, reduced in our animal model, have anti-inflammatory properties [67,68], possibly emphasizing their role in the pathophysiology of NASH. Furthermore, this specific family can directly increase the single- and multi-unit firing rates of the mesenteric nerve bundle and decrease anxiety/depression in mice [69,70], linking microbiota dysbiosis to changes in mood and behavior [71].

As expected from the differences observed in the composition of the intestinal microbiota, the levels of the main intestinal microbial metabolites, the SCFAs, also differed between the groups, with lower levels of the main SCFAs (acetate, propionate, and butyrate) in the NASH animals. The production of these metabolites was found to be linked to microorganisms from the Proteobacteria, Firmicutes, and Bacteroidetes phyla. In and of themselves, since anti-inflammatory properties have been attributed to acetate [72], propionate [72,73] and butyrate [74,75], microbial-derived SCFAs are also involved in inflammatory modulation [76]. In addition, SCFAs have also increasingly been implicated in emotional processing and behaviors [77].

In this regard, the NASH group showed depressive-like behavior, as measured in the forced swimming test. The NASH group spent significantly less time swimming and more time immobile than the NC group, demonstrating a sense of hopelessness as reflected in the lack of struggle to escape the cylinder [78,79]. However, the NASH group did not display anxious behaviors. Some studies have shown that high-cholesterol [80] and high-fat diets [81] lead to anxious-like behavior, whereas other studies have found an anxiolytic effect [82,83]. Thus, future studies should focus on how the type and length of the diet administered, along with the age of the animals, could influence these behaviors.

Moreover, it has been suggested that NASH-associated cognitive impairment is mediated by insulin resistance, as demonstrated in animal models [79,84]. Insulin resistance is associated with impaired glucose utilization and lipid metabolism, resulting in increased oxidative stress and inflammation [85]. In addition, insulin resistance has also been linked to gut dysbiosis and altered SCFA secretion [86].

Although this study did not measure insulin resistance, it has been previously reported that this model showed no differences in insulin and the HOMA index, whereas there was an increase in total IGF-I (insulin growth factor-I) [6]. Some studies have shown that IGF-I is increased in affective disorders [87,88], highlighting its role as a mood regulator [89,90], for example through its potent anxiolytic actions [91]. Therefore, the absence of anxiety-like behavior, along with the increased IGF-I, may point to an endogenous protective mechanism [92] in our NASH model.

Depression is not only associated with a reduction in quality of life, but is also often accompanied by other symptoms, such as cognitive impairment, functional impairment, and social dysfunction [84]. Because depression is related to social dysfunction, short-term social memory was studied. Our results reveal that NASH animals were not interested in exploring new intruders (social component), reflecting an impairment in social behavior.

With regards to the other cognitive functions, we found that the NASH group had, according to the results of two independent tests, preserved spatial reference memory and a hippocampal-dependent long-term type of memory. Conflicting results have been observed as a consequence of different diets. As such, Ross et al. [93] found that fructose-induced fatty liver disease is associated with a deficit in this specific type of memory, confirmed by Darling et al. [94] in animals that voluntarily consumed high-energy diets with elevated amounts of fat and sugar. Furthermore, Hargrave et al. [95] found changes in the use of hippocampal-dependent strategies depending on the obesogenic properties of the high-fat high-sugar Western diet provided. In addition, this deficit was also associated with high-fat and obesogenic diets in studies by Boitard et al. [96], Wang et al. [97], and Spencer et al. [98].

Although we did not observe a deficit in long-term memory in the NASH model, we did observe changes in short-term memory, as assessed by evaluating spatial working memory, which is mainly dependent on the prefrontal cortex. Our results showed that the NASH group had difficulty remembering a particular spatial location for a short period of time. This impairment was not caused by problems in locomotor activity related to the pathology because, as previously described in high fat [99] and high cholesterol diets [100], they showed no differences in motor coordination or resistance to fatigue compared to the NC group. Previous studies showed that prolonged administration of a high saturated fat diet was found to impair the performance of young and old rats on the RAWM [14], as well in tests of working memory [101]. However, Deshpande et al. [12] did not observe any working memory deficits when old animals were treated with the same diet for shorter periods (8 weeks), whereas Granholm et al. [13] did find working memory deficits when this diet was accompanied with elevated cholesterol levels.

In order to clarify the mechanisms triggering this cognitive deficit, we measured brain metabolic activation and monoamine determination resulting from the execution of the spatial working memory task. It has been reported that ceramides and other toxic lipids generated by the liver in the context of NASH pathology could mediate the adverse effects on the brain due to their capacity to cross the blood-brain barrier and, once inside the brain, cause neuroinflammation, oxidative stress, metabolic impairment, and neurotransmitter deficits [102]. Moreover, several studies have shown that SCFAs are also used as a mitochondrial energy source in both humans and rodents [103–105]. Indeed, relevant levels of acetate and propionate have been reported to directly influence the brain [106,107].

NAFLD pathophysiology has been found to involve mitochondrial dysfunction linked to oxidative stress and altered oxidative phosphorylation [108]. Therefore, neural metabolism was measured by labelling CCO, a mitochondrial enzyme involved in ATP production. This method was previously used to clarify the cerebral oxidative energetic metabolic changes underlying learning impairments in liver disease [109,110]. The NASH group had lower levels of CCO activation in the prefrontal cortex, hippocampus, thalamus, amygdala, and mammillary bodies, which form part of a network underlying spatial working memory. The prefrontal cortex has traditionally been pointed out as the region modulating spatial working memory [111,112], although it has also been shown that it needs to be synchronized with the hippocampus to this end [113,114]. The thalamus has been proposed as a key node in this communication line between the prefrontal cortex and hippocampus [115,116], as well as with the amygdala [111,116] and the mammillary bodies [117]. Thus, the decreased CCO activation in these areas, reflecting a mitochondrial alteration, may explain the animals’ impaired performance on the task. Brain mitochondrial dysfunction is one of the main forces driving neurodegeneration [118], and to our knowledge, had not been previously described in NASH.

Furthermore, neuronal communication requires a high amount of energy, which is reduced in the NASH group, and is critically related to cellular metabolism and energy supply, both dependent on glucose availability [119]. Our results suggest an imbalance in glucose metabolism, which has been found to affect neuronal circuits and be especially relevant to prefrontal functioning since the prefrontal cortex has a high density of excitatory synapses [120] whose activity is strictly dependent on glucose availability [121]. These prefrontal cortical excitatory synapses have been associated with DA because this monoamine can modulate the response of glutamatergic pyramidal neurons in this region [122]. Accordingly, we not only found reduced levels of DA in the prefrontal cortex but also an increased DOPAC/DA ratio in the cerebellum in the NASH group, representing enhanced DA catabolism.

Dopaminergic dysfunction and reduced mitochondrial oxidative activity could have been altered as a consequence of insulin resistance, potentially through altered expression of monoamine oxidase and electron transport chain proteins [123]. The dopaminergic neurons’ acid-sensitive ionic channels, which have been found to be sensitive to ammonia [124], may potentially explain the DA dysregulation observed in our NASH model. Exposure to this substance has been associated with a reduction in DA levels in a dose-dependent manner, along with an increased DOPAC/DA ratio, probably because monoaminergic enzymatic regulation is affected [125]. Consequently, these alterations could be derived from the hyperammonemia [126] observed in our NASH group, which itself may be a consequence of the observed gut dysbiosis [63]. Moreover, intestinal bacteria and SCFAs are known to influence DA and NA levels [127,128]. Therefore, the gut dysbiosis and decreased production of SCFAs found in NASH animals, acting through the gut-brain axis, could also be responsible for the DA depletion.

The mesolimbic DA system, and, specifically, its signaling in the prefrontal cortex, plays an important role in depression [129,130]. This may explain the differences found in the NASH group. DA modulation in the prefrontal cortex is also related to social behavior [122,131] and is essential for modulating cognitive functions such as working memory [132]. Moreover, dopaminergic projections from the prefrontal cortex extend out to the amygdala [133], the hippocampus [134], and the cerebellum [135]. Indeed, dopaminergic modulation in the cerebellum is also involved in modulating cognitive functions such as social recognition [136], as reflected in the NASH group rats’ lack of interest in the social context, and working memory [137], which is also disturbed in this pathology. Li et al. [138] showed that mRNA expression of tyrosine hydroxylase, as the rate-limiting step in dopamine synthesis, is decreased in animals fed a high-fat diet. However, it has never been reported whether high-fat high-cholesterol induced-NASH could cause dopaminergic neuron injury. In addition, dopaminergic neurocircuitry may be altered as a consequence of an increased inflammatory response [139], which could be a potential mechanism underlying the DA deficits in our NASH model. Our study demonstrated for the first time that this type of diet led to a decrease in DA in the prefrontal cortex alongside a decrease in its turnover in the cerebellum. These changes were associated with an increase in immobility in the forced swimming test and alterations in the cognitive functions driven specifically by these brain regions. Finally, the thalamus and mammillary bodies have been reported to play a role in DA regulation [140,141], and decreased NA levels have been found in the striatum of the NASH group. The striatum is known to not only be involved in this dopaminergic pathway, but also to be modulated by noradrenergic signaling [142], and a reduction in NA in the striatum has been associated with depressive-like behavior [143]. Moreover, as was the case with DA, NA levels have been also shown to be reduced in a dose-dependent manner as a consequence of exposure to ammonia [125].

Many clinical observations highlight the importance of considering the interactions between the dopaminergic, serotoninergic, and noradrenergic systems. In this regard, our results showed no changes in serotonergic systems. This may either be due to these neurons’ acid-sensitive ion channels not being specifically sensitive to ammonia, or to a balance between the metabolic disruption of ammonia-modulated monoaminergic anabolism [125] and the increased transport of tryptophan across the brain-blood barrier as a result of ammonia exposure [144,145].

In conclusion, these results indicate that a high-fat, high-cholesterol-induced NASH-like condition produces gut dysbiosis and decreases gut microbial SCFA production, while enhancing hyperammonemia. These findings may suggest that gut-derived microbiota metabolites, along with ammonia, may be generating a neurotoxic injury that could be reflected not only in behavioral changes, but also in metabolic and functional brain regional deficits. However, more studies are warranted to fully understand the mechanisms underlying the functional changes resulting from high-fat and high cholesterol induced cognitive impairment. In addition, further studies will also be necessary to determine the role of inflammation in the cognitive decline following gut microbe dysbiosis.

## Supporting information

S1 Table Relative abundances at genus level of microbial groups in NC and NASH subjects (*p<0.05). (XLSX)

## References

[1] Tomic D, Kemp WW, Roberts SK. Nonalcoholic fatty liver disease: current concepts, epidemiology and management strategies. Eur J Gastroenterol Hepatol. 2018;30(10):1103–15. 10.1097/MEG.0000000000001235 30113367

[2] Carr RM, Oranu A, Khungar V. Non-alcoholic fatty liver disease: pathophysiology and management. Gastroenterol Clin North Am. 2016;45(4):639–52. 10.1016/j.gtc.2016.07.003 27837778 PMC5127277

[3] Farrell GC, Larter CZ. Nonalcoholic fatty liver disease: from steatosis to cirrhosis. Hepatology. 2006;43(2 Suppl 1):S99–112. 10.1002/hep.20973 16447287

[4] Carter-Kent C, Zein NN, Feldstein AE. Cytokines in the pathogenesis of fatty liver and disease progression to steatohepatitis: implications for treatment. Am J Gastroenterol. 2008;103(4):1036–42. 18177455 10.1111/j.1572-0241.2007.01709.x

[5] Santhekadur PK, Kumar D, Sanyal A. Preclinical models of nonalcoholic fatty liver. J Hepatol. 2018;68(2):230–7. 10.1016/j.jhep.2017.10.031 29128391 PMC5775040

[6] Thomsen KL, Grønbæk H, Glavind E, Hebbard L, Jessen N, Clouston A, et al Experimental nonalcoholic steatohepatitis compromises ureagenesis, an essential hepatic metabolic function. Am J Physiol Gastrointest Liver Physiol. 2014;307(3):G295–301. 10.1152/ajpgi.00036.2014 24924745

[7] De Chiara F, Heebøll S, Marrone G, Montoliu C, Hamilton-Dutoit S, Ferrandez A, et al Urea cycle dysregulation in non-alcoholic fatty liver disease. J Hepatol. 2018;69(4):905–15. 10.1016/j.jhep.2018.06.023 29981428

[8] Christensen CU, Glavind E, Thomsen KL, Kim YO, Heebøll S, Schuppan D, et al Niemann-Pick type C2 protein supplementation in experimental nonalcoholic fatty liver disease. PLoS One. 2018;13(3):e0192728 10.1371/journal.pone.0192728 29522534 PMC5844539

[9] Heebøll S, Thomsen KL, Clouston A, Sundelin EI, Radko Y, Christensen LP, et al Effect of resveratrol on experimental non-alcoholic steatohepatitis. Pharmacol Res. 2015;95–96:34–41. 10.1016/j.phrs.2015.03.005 25814186

[10] Thomsen KL, Aagaard NK, Grønbæk H, Holst JJ, Jessen N, Frystyk J, et al IL-6 has no acute effect on the regulation of urea synthesis in vivo in rats. Scand J Clin Lab Invest. 2011;71(2):150–6. 10.3109/00365513.2010.547213 21190512

[11] Morris MC, Tangney CC, Wang Y, Sacks FM, Barnes LL, Bennett DA, et al MIND diet slows cognitive decline with aging. Alzheimer’s Dement. 2015;11(9):1015–22. 26086182 10.1016/j.jalz.2015.04.011 PMC4581900

[12] Deshpande NG, Saxena J, Pesaresi TG, Carrell CD, Ashby GB, Liao M-K, et al High fat diet alters gut microbiota but not spatial working memory in early middle-aged Sprague Dawley rats. PLoS One. 2019;14(5):e0217553 10.1371/journal.pone.0217553 31141574 PMC6541285

[13] Granholm A-C, Bimonte-Nelson HA, Moore AB, Nelson ME, Freeman LR, Sambamurti K. Effects of a saturated fat and high cholesterol diet on memory and hippocampal morphology in the middle-aged rat. J Alzheimer’s Dis. 2008;14(2):133–45. 18560126 10.3233/jad-2008-14202 PMC2670571

[14] Ledreux A, Wang X, Schultzberg M, Granholm AC, Freeman LR. Detrimental effects of a high fat/high cholesterol diet on memory and hippocampal markers in aged rats. Behav Brain Res. 2016;312:294–304. 10.1016/j.bbr.2016.06.012 27343935 PMC7388174

[15] Stam R, Akkermans LMA, Wiegant VM. Trauma and the gut: interactions between stressful experience and intestinal function. Gut. 1997;40(6):704–9. 10.1136/gut.40.6.704 9245921 PMC1027192

[16] Klooker TK, Braak B, Painter RC, de Rooij SR, van Elburg RM, van den Wijngaard RM, et al Exposure to severe wartime conditions in early life is associated with an increased risk of irritable bowel syndrome: a population-based cohort study. Am J Gastroenterol. 2009;104(9):2250–6. 19513027 10.1038/ajg.2009.282

[17] Moloney RD, Johnson AC, O’Mahony SM, Dinan TG, Greenwood-Van Meerveld B, Cryan JF. Stress and the microbiota-gut-brain axis in visceral pain: relevance to irritable bowel syndrome. CNS Neurosci Ther. 2016;22(2):102–17. 10.1111/cns.12490 26662472 PMC6492884

[18] Bharwani A, Mian MF, Foster JA, Surette MG, Bienenstock J, Forsythe P. Structural & functional consequences of chronic psychosocial stress on the microbiome & host. Psychoneuroendocrinology. 2016;63:217–27. 10.1016/j.psyneuen.2015.10.001 26479188

[19] De Vadder F, Kovatcheva-Datchary P, Goncalves D, Vinera J, Zitoun C, Duchampt A, et al Microbiota-generated metabolites promote metabolic benefits via gut-brain neural circuits. Cell. 2014;156(1–2):84–96. 10.1016/j.cell.2013.12.016 24412651

[20] Li Z, Yi CX, Katiraei S, Kooijman S, Zhou E, Chung CK, et al Butyrate reduces appetite and activates brown adipose tissue via the gut-brain neural circuit. Gut. 2018;67(7):1269–79. 10.1136/gutjnl-2017-314050 29101261

[21] Chambers ES, Viardot A, Psichas A, Morrison DJ, Murphy KG, Zac-Varghese SEK, et al Effects of targeted delivery of propionate to the human colon on appetite regulation, body weight maintenance and adiposity in overweight adults. Gut. 2015;64(11):1744–54. 10.1136/gutjnl-2014-307913 25500202 PMC4680171

[22] Reigstad CS, Salmonson CE, Rainey JF, Szurszewski JH, Linden DR, Sonnenburg JL, et al Gut microbes promote colonic serotonin production through an effect of short-chain fatty acids on enterochromaffin cells. FASEB J. 2015;29(4):1395–403. 10.1096/fj.14-259598 25550456 PMC4396604

[23] Agrawal R, Noble E, Vergnes L, Ying Z, Reue K, Gomez-Pinilla F. Dietary fructose aggravates the pathobiology of traumatic brain injury by influencing energy homeostasis and plasticity. J Cereb Blood Flow Metab. 2016;36(5):941–53. 10.1177/0271678X15606719 26661172 PMC4853835

[24] Mastrocola R, Nigro D, Cento AS, Chiazza F, Collino M, Aragno M. High-fructose intake as risk factor for neurodegeneration: key role for carboxy methyllysine accumulation in mice hippocampal neurons. Neurobiol Dis. 2016;89:65–75. 10.1016/j.nbd.2016.02.005 26851500

[25] Rojas JC, Bruchey AK, Gonzalez-Lima F. Low-level light therapy improves cortical metabolic capacity and memory retention. J Alzheimer’s Dis. 2012;32(3):741–52. 22850314 10.3233/JAD-2012-120817

[26] Wouters K, van Gorp PJ, Bieghs V, Gijbels MJ, Duimel H, Lütjohann D, et al Dietary cholesterol, rather than liver steatosis, leads to hepatic inflammation in hyperlipidemic mouse models of nonalcoholic steatohepatitis. Hepatology. 2008;48(2):474–86. 10.1002/hep.22363 18666236

[27] Ikemoto S, Takahashi M, Tsunoda N, Maruyama K, Itakura H, Kawanaka K, et al Cholate inhibits and obesity with acyl-CoA synthetase hyperglycemia mRNA decrease. Am J Physiol. 1997;273(1 Pt 1):E37–45. 9252477 10.1152/ajpendo.1997.273.1.E37

[28] Kucera O, Cervinkova Z. Experimental models of non-alcoholic fatty liver disease in rats. World J Gastroenterol. 2014;20(26):8364–76. 10.3748/wjg.v20.i26.8364 25024595 PMC4093690

[29] Arboleya S, Binetti A, Salazar N, Ferna N, Solís G, Hernández-Barranco A, et al Establishment and development of intestinal microbiota in preterm neonates. FEMS Microbiol Ecol. 2012;79(3):763–72. 10.1111/j.1574-6941.2011.01261.x 22126419

[30] Milani C, Hevia A, Foroni E, Duranti S, Turroni F, Gueimonde M, et al Assessing the fecal microbiota: an optimized ion torrent 16S rRNA gene-based analysis protocol. PLoS One. 2013;8(7):e68739 10.1371/journal.pone.0068739 23869230 PMC3711900

[31] Nogacka A, Salazar N, Suárez M, Milani C, Arboleya S, Solís G, et al Impact of intrapartum antimicrobial prophylaxis upon the intestinal microbiota and the prevalence of antibiotic resistance genes in vaginally delivered full-term neonates. Microbiome. 2017;5(1):93 10.1186/s40168-017-0313-3 28789705 PMC5549288

[32] Edgar RC. Search and clustering orders of magnitude faster than BLAST. Bioinformatics. 2010;26(19):2460–1. 10.1093/bioinformatics/btq461 20709691

[33] Quast C, Pruesse E, Yilmaz P, Gerken J, Schweer T, Glo FO, et al The SILVA ribosomal RNA gene database project: improved data processing and web-based tools. Nucleic Acids Res. 2013;41:D590–596. 10.1093/nar/gks1219 23193283 PMC3531112

[34] Arboleya S, Sánchez B, Ventura M, Margolles A, Fernández N, de los Reyes-Gavilán CG, et al Intestinal microbiota development in preterm neonates and effect of perinatal antibiotics. J Pediatr. 2015;166(3):538–44. 10.1016/j.jpeds.2014.09.041 25444008

[35] Valdés L, Salazar N, González S, Arboleya S, Ríos-Covián D, Genovés S, et al Selection of potential probiotic bifidobacteria and prebiotics for elderly by using in vitro faecal batch cultures. Eur Food Res Technol. 2017;243(1):157–65.

[36] Moris G, Arboleya S, Mancabelli L, Milan C, Ventura M, Re CGDL, et al Fecal microbiota profile in a group of myasthenia gravis patients. Sci Rep. 2018;8(1):14384 10.1038/s41598-018-32700-y 30258104 PMC6158187

[37] Jones BJ, Roberts DJ. The quantitative measurement of motor inco-ordination in naive mice using an accelerating rotarod. J Pharm Pharmacol. 1968;20(4):302–4. 10.1111/j.2042-7158.1968.tb09743.x 4384609

[38] Slattery DA, Markou A, Cryan JF. Evaluation of reward processes in an animal model of depression. Psychopharmacology (Berl). 2007;190(4):555–68. 17177055 10.1007/s00213-006-0630-x

[39] Porsolt RD, Anton G, Blavet N, Jalfre M. Behavioural despair in rats: a new model sensitive to antidepressant treatments. Eur J Pharmacol. 1978;47(4):379–91. 10.1016/0014-2999(78)90118-8 204499

[40] Prado VF, Martins-Silva C, de Castro BM, Lima RF, Barros DM, Amaral E, et al Mice deficient for the vesicular acetylcholine transporter are myasthenic and have deficits in object and social recognition. Neuron. 2006;51(5):601–12. 10.1016/j.neuron.2006.08.005 16950158

[41] Méndez M, Méndez-López M, López L, Aller MA, Arias J, Arias JL. Working memory impairment and reduced hippocampal and prefrontal cortex c-Fos expression in a rat model of cirrhosis. Physiol Behav. 2008;95(3):302–7. 10.1016/j.physbeh.2008.06.013 18634813

[42] Gonzalez-Lima F, Cada A. Cytochrome oxidase activity in the auditory system of the mouse: a qualitative and quantitative histochemical study. Neuroscience. 1994;63(2):559–78. 10.1016/0306-4522(94)90550-9 7891865

[43] Wong-Riley MTT. Cytochrome oxidase: an endogenous metabolic marker for neuron activity. Trends Neurosci. 1989;12(3):94–101. 10.1016/0166-2236(89)90165-3 2469224

[44] González-Pardo H, Novelli A, Menéndez-Patterson A, Arias JL. The developmental of oxidative metabolism in diencephalic structures of the rat: a quantitative study. Brain Res Bull. 1996;41(1):31–8. 10.1016/0361-9230(96)00007-x 8883913

[45] Paxinos G, Watson C. The rat brain in stereotaxic coordinates. 5th ed Elsevier Academic Press; 2004.

[46] Francque S, Verrijken A, Mertens I, Hubens G, Van Marck E, Pelckmans P, et al Noncirrhotic human nonalcoholic fatty liver disease induces portal hypertension in relation to the histological degree of steatosis. Eur J Gastroenterol Hepatol. 2010;22(12):1449–57. 21389796 10.1097/MEG.0b013e32833f14a1

[47] Marques C, Meireles M, Norberto S, Leite J, Freitas J, Pestana D, et al High-fat diet-induced obesity rat model: a comparison between Wistar and Sprague-Dawley rat. Adipocyte. 2016;5(1):11–21. 10.1080/21623945.2015.1061723 27144092 PMC4836488

[48] Xu ZJ, Fan JG, Ding XD, Qiao L, Wang GL. Characterization of high-fat, diet-induced, non-alcoholic steatohepatitis with fibrosis in rats. Dig Dis Sci. 2010;55(4):931–40. 10.1007/s10620-009-0815-3 19459046 PMC2946554

[49] Byrne CD, Targher G. NAFLD: a multisystem disease. J Hepatol. 2015;62(1 Suppl):S47–64. 10.1016/j.jhep.2014.12.012 25920090

[50] Yki-Järvinen H. Diagnosis of non-alcoholic fatty liver disease (NAFLD). Diabetologia. 2016;59(6):1104–11. 10.1007/s00125-016-3944-1 27091184

[51] Neuman MG, Cohen LB, Nanau RM. Biomarkers in nonalcoholic fatty liver disease. Can J Gastroenterol Hepatol. 2014;28(11):607–18. 25575111 10.1155/2014/757929 PMC4277175

[52] Skowrońska M, Albrecht J. Alterations of blood brain barrier function in hyperammonemia: an overview. Neurotox Res. 2012;21(2):236–44. 10.1007/s12640-011-9269-4 21874372 PMC3246587

[53] Hernández-Rabaza V, Cabrera-Pastor A, Taoro-González L, Malaguarnera M, Agustí A, Llansola M, et al Hyperammonemia induces glial activation, neuroinflammation and alters neurotransmitter receptors in hippocampus, impairing spatial learning: reversal by sulforaphane. J Neuroinflammation. 2016;13:41 10.1186/s12974-016-0505-y 26883214 PMC4754839

[54] Rodrigo R, Cauli O, Gomez-Pinedo U, Agusti A, Hernandez-Rabaza V, Garcia-Verdugo JM, et al Hyperammonemia induces neuroinflammation that contributes to cognitive impairment in rats with hepatic encephalopathy. Gastroenterology. 2010;139(2):675–84. 10.1053/j.gastro.2010.03.040 20303348

[55] Freeman LR, Zhang L, Nair A, Dasuri K, Francis J, Fernandez-Kim S-O, et al Obesity increases cerebrocortical reactive oxygen species and impairs brain function. Free Radic Biol Med. 2013;56:226–33. 10.1016/j.freeradbiomed.2012.08.577 23116605 PMC4038352

[56] Stranahan AM, Norman ED, Lee K, Cutler RG, Telljohann R, Egan JM, et al Diet-induced insulin resistance impairs hippocampal synaptic plasticity and cognition in middle-aged rats. Hippocampus. 2008;18(11):1085–8. 10.1002/hipo.20470 18651634 PMC2694409

[57] Diaz Heijtz R, Wang S, Anuar F, Qian Y, Björkholm B, Samuelsson A, et al Normal gut microbiota modulates brain development and behavior. Proc Natl Acad Sci U S A. 2011;108(7):3047–52. 10.1073/pnas.1010529108 21282636 PMC3041077

[58] Neufeld KM, Kang N, Bienenstock J, Foster JA. Reduced anxiety-like behavior and central neurochemical change in germ-free mice. Neurogastroenterol Motil. 2011;23(3):255–65. 10.1111/j.1365-2982.2010.01620.x 21054680

[59] Bercik P, Denou E, Collins J, Jackson W, Lu J, Jury J, et al The intestinal microbiota affect central levels of brain-derived neurotropic factor and behavior in mice. Gastroenterology. 2011;141(2):599–609. 10.1053/j.gastro.2011.04.052 21683077

[60] Turnbaugh PJ, Ley RE, Mahowald MA, Magrini V, Mardis ER, Gordon JI. An obesity-associated gut microbiome with increased capacity for energy harvest. Nature. 2006;444(7122):1027–31. 10.1038/nature05414 17183312

[61] Turnbaugh PJ, Backhed F, Fulton L, Gordon JI. Marked alterations in the distal gut microbiome linked to diet-induced obesity. Cell Host Microbe. 2008;3(4):213–23. 18407065 10.1016/j.chom.2008.02.015 PMC3687783

[62] Bruce-Keller AJ, Salbaum JM, Luo M, Iv EB, Taylor CM, Welsh DA, et al Obese-type gut microbiota induce neurobehavioral changes in the absence of obesity. Biol Psychiatry. 2015;77(7):607–15. 10.1016/j.biopsych.2014.07.012 25173628 PMC4297748

[63] Shawcross DL. Is it time to target gut dysbiosis and immune dysfunction in the therapy of hepatic encephalopathy? Expert Rev Gastroenterol Hepatol. 2015;9(5):539–42. 10.1586/17474124.2015.1035257 25846450

[64] Kang DJ, Betrapally NS, Gosh SA, Sartor RB, Hylemon PB, Gillevet PM, et al Gut microbiota drive the development of neuro-inflammatory response in cirrhosis. Hepatology. 2016;64(4):1232–48. 10.1002/hep.28696 27339732 PMC5033692

[65] Boulangé CL, Neves AL, Chilloux J, Nicholson JK, Dumas ME. Impact of the gut microbiota on inflammation, obesity, and metabolic disease. Genome Med. 2016;8(1):42 10.1186/s13073-016-0303-2 27098727 PMC4839080

[66] Ma J, Zhou Q, Li H. Gut microbiota and nonalcoholic fatty liver disease: insights on mechanisms and therapy. Nutrients. 2017;9(10). 10.3390/nu9101124 PMC5691740 29035308

[67] Plaza-Díaz J, Ruiz-Ojeda FJ, Vilchez-Padial LM, Gil A. Evidence of the anti-inflammatory effects of probiotics and synbiotics in intestinal chronic diseases. Nutrients. 2017;9(6). 10.3390/nu9060555 PMC5490534 28555037

[68] Nagpal R, Kumar M, Yadav AK, Hemalatha R, Yadav H, Marotta F, et al Gut microbiota in health and disease: an overview focused on metabolic inflammation. Benef Microbes. 2016;7(2):181–94. 10.3920/bm2015.0062 26645350

[69] Bravo JA, Forsythe P, Chew M V., Escaravage E, Savignac HM, Dinan TG, et al Ingestion of Lactobacillus strain regulates emotional behavior and central GABA receptor expression in a mouse via the vagus nerve. Proc Natl Acad Sci U S A. 2011;108(38):16050–5. 10.1073/pnas.1102999108 21876150 PMC3179073

[70] Perez-Burgos A, Wang B, Mao Y-K, Mistry B, Neufeld K-AM, Bienenstock J, et al Psychoactive bacteria Lactobacillus rhamnosus (JB-1) elicits rapid frequency facilitation in vagal afferents. Am J Physiol Liver Physiol. 2013;304(2):G211–20. 10.1152/ajpgi.00128.2012 23139216

[71] Dinan TG, Cryan JF. Melancholic microbes: a link between gut microbiota and depression? Neurogastroenterol Motil. 2013;25(9):713–9. 10.1111/nmo.12198 23910373

[72] Tedelind S, Westberg F, Kjerrulf M, Vidal A. Anti-inflammatory properties of the short-chain fatty acids acetate and propionate: a study with relevance to inflammatory bowel disease. World J Gastroenterol. 2007;13(20):2826–32. 10.3748/wjg.v13.i20.2826 17569118 PMC4395634

[73] Al-Lahham SH, Roelofsen H, Priebe M, Weening D, Dijkstra M, Hoek A, et al Regulation of adipokine production in human adipose tissue by propionic acid. Eur J Clin Invest. 2010;40(5):401–7. 10.1111/j.1365-2362.2010.02278.x 20353437

[74] Fukae J, Amasaki Y, Yamashita Y, Bohgaki T, Yasuda S, Jodo S, et al Butyrate suppresses tumor necrosis factor α production by regulating specific messenger RNA degradation mediated through a cis-acting AU-rich element. Arthritis Rheum. 2005;52(9):2697–707. 10.1002/art.21258 16142751

[75] Säemann MD, Böhmig GA, Österreicher CH, Burtscher H, Parolini O, Diakos C, et al Anti-inflammatory effects of sodium butyrate on human monocytes: potent inhibition of IL-12 and up-regulation of IL-10 production. FASEB J. 2000;14(15):2380–2. 10.1096/fj.00-0359fje 11024006

[76] Saad MJA, Santos A, Prada PO. Linking gut microbiota and inflammation to obesity and insulin resistance. Physiology. 2016;31(4):283–93. 10.1152/physiol.00041.2015 27252163

[77] van de Wouw M, Boehme M, Lyte JM, Wiley N, Strain C, O’Sullivan O, et al Short-chain fatty acids: microbial metabolites that alleviate stress-induced brain–gut axis alterations. J Physiol. 2018;596(20):4923–44. 10.1113/JP276431 30066368 PMC6187046

[78] Bogdanova O V, Kanekar S, Renshaw PF. Factors influencing behavior in the forced swim test. Physiol Behav. 2017;118:227–39. 10.1016/j.physbeh.2013.05.012 PMC5609482 23685235

[79] Mebel DM, Wong JCY, Dong YJ, Borgland SL. Insulin in the ventral tegmental area reduces hedonic feeding and suppresses dopamine concentration via increased reuptake. Eur J Neurosci. 2012;36(3):2336–46. 10.1111/j.1460-9568.2012.08168.x 22712725 PMC5239666

[80] Strekalova T, Evans M, Costa-Nunes J, Bachurin S, Yeritsyan N, Couch Y, et al Tlr4 upregulation in the brain accompanies depression- and anxiety-like behaviors induced by a high-cholesterol diet. Brain Behav Immun. 2015;48:42–7. 10.1016/j.bbi.2015.02.015 25712260

[81] Del Rosario A, McDermott MM, Panee J. Effects of high fat diet and bamboo extract supplement on anxiety- an depression-like neurobehaviors in mice. Br J Nutr. 2015;33(4):395–401. 10.1017/S0007114511006738 PMC4659481 22313665

[82] McNeilly AD, Stewart CA, Sutherland C, Balfour DJK. High fat feeding is associated with stimulation of the hypothalamic-pituitary-adrenal axis and reduced anxiety in the rat. Psychoneuroendocrinology. 2015;52:272–80. 10.1016/j.psyneuen.2014.12.002 25544739

[83] Prasad A, Prasad C. Short-term consumption of a diet rich in fat decreases anxiety response in adult male rats. Physiol Behav. 1996;60(3):1039–42. 10.1016/0031-9384(96)00135-7 8873290

[84] Gonda X, Pompili M, Serafini G, Carvalho AF, Rihmer Z, Dome P. The role of cognitive dysfunction in the symptoms and remission from depression. Ann Gen Psychiatry. 2015;14:27 10.1186/s12991-015-0068-9 26396586 PMC4578787

[85] Tangvarasittichai S. Oxidative stress, insulin resistance, dyslipidemia and type 2 diabetes mellitus. World J Diabetes. 2015;6(3):456 10.4239/wjd.v6.i3.456 25897356 PMC4398902

[86] Caricilli AM, Saad MJA. The role of gut microbiota on insulin resistance. Nutrients. 2013;5(3):829–51. 10.3390/nu5030829 23482058 PMC3705322

[87] Deuschle M, Blum WF, Strasburger CJ, Weber B, Krner A, Standhardt H, et al Insulin-like growth factor (IGF-I) plasma concentrations are increased in depressed patients. Psychoneuroendocrinology. 1997;22(7):493–503. 10.1016/s0306-4530(97)00046-2 9373883

[88] Bot M, Milaneschi Y, Penninx BWJH, Drent ML. Plasma insulin-like growth factor I levels are higher in depressive and anxiety disorders, but lower in antidepressant medication users. Psychoneuroendocrinology. 2016;68:148–55. 10.1016/j.psyneuen.2016.02.028 26974499

[89] Baldini S, Restani L, Baroncelli L, Coltelli M, Franco R, Cenni MC, et al Enriched early life experiences reduce adult anxiety-like behavior in rats: a role for insulin-like growth factor 1. J Neurosci. 2013;33(28):11715–23. 10.1523/JNEUROSCI.3541-12.2013 23843538 PMC6618685

[90] Mitschelen M, Yan H, Farley JA, Warrington JP, Han S, Hereñú CB, et al Long-term deficiency of circulating and hippocampal insulin-like growth factor I induces depressive behavior in adult mice: a potential model of geriatric depression. Neuroscience. 2011;185:50–60. 10.1016/j.neuroscience.2011.04.032 21524689 PMC3101268

[91] Hoshaw BA, Hill TI, Crowley JJ, Malberg JE, Khawaja X, Rosenzweig-Lipson S, et al Antidepressant-like behavioral effects of IGF-I produced by enhanced serotonin transmission. Eur J Pharmacol. 2008;594(1–3):109–16. 10.1016/j.ejphar.2008.07.023 18675266 PMC2719710

[92] Santi A, Bot M, Aleman A, Penninx BWJH, Aleman IT. Circulating insulin-like growth factor I modulates mood and is a biomarker of vulnerability to stress: from mouse to man. Transl Psychiatry. 2018;8(1):142 10.1038/s41398-018-0196-5 30068974 PMC6070549

[93] Ross AP, Bruggeman EC, Kasumu AW, Mielke JG, Parent MB. Non-alcoholic fatty liver disease impairs hippocampal-dependent memory in male rats. Physiol Behav. 2012;106(2):133–41. 10.1016/j.physbeh.2012.01.008 22280920

[94] Darling JN, Ross AP, Bartness TJ, Parent MB. Predicting the effects of a high-energy diet on fatty liver and hippocampal-dependent memory in male rats. Obesity. 2013;21(5):910–7. 10.1002/oby.20167 23784893 PMC3695417

[95] Hargrave SL, Davidson TL, Zheng W, Kinzig KP. Western diets induce blood-brain barrier leakage and alter spatial strategies in rats. Behav Neurosci. 2016;130(1):123–35. 10.1037/bne0000110 26595878 PMC4795941

[96] Boitard C, Cavaroc A, Sauvant J, Aubert A, Castanon N, Layé S, et al Impairment of hippocampal-dependent memory induced by juvenile high-fat diet intake is associated with enhanced hippocampal inflammation in rats. Brain Behav Immun. 2014;40:9–17. 10.1016/j.bbi.2014.03.005 24662056

[97] Wang D, Yan J, Chen J, Wu W, Zhu X, Wang Y. Naringin improves neuronal insulin signaling, brain mitochondrial function, and cognitive function in high-fat diet-induced obese mice. Cell Mol Neurobiol. 2015;35(7):1061–71. 10.1007/s10571-015-0201-y 25939427 PMC11486290

[98] Spencer SJ, D’Angelo H, Soch A, Watkins LR, Maier SF, Barrientos RM. High-fat diet and aging interact to produce neuroinflammation and impair hippocampal- and amygdalar-dpendent memory. Neurobiol Aging. 2017;58:88–101. 10.1016/j.neurobiolaging.2017.06.014 28719855 PMC5581696

[99] Pintana H, Apaijai N, Pratchayasakul W, Chattipakorn N, Chattipakorn SC. Effects of metformin on learning and memory behaviors and brain mitochondrial functions in high fat diet induced insulin resistant rats. Life Sci. 2012;91(11–12):409–14. 10.1016/j.lfs.2012.08.017 22925597

[100] Hu X, Wang T, Luo J, Liang S, Li W, Wu X, et al Age-dependent effect of high cholesterol diets on anxiety-like behavior in elevated plus maze test in rats. Behav Brain Funct. 2014;10:30 10.1186/1744-9081-10-30 25179125 PMC4158000

[101] Wang Z, Fan J, Wang J, Li Y, Xiao L, Duan D, et al Protective effect of lycopene on high-fat diet-induced cognitive impairment in rats. Neurosci Lett. 2016;627:185–91. 10.1016/j.neulet.2016.05.014 27177726

[102] de la Monte SM, Longato L, Tong M, Wands JR. Insulin resistance and neurodegeneration: roles of obesity, type 2 diabetes mellitus and non-alcoholic steatohepatitis. Curr Opin Investig Drugs. 2009;10(10):1049–60. 19777393 PMC4600072

[103] Egorin MJ, Yuan ZM, Sentz DL, Plaisance K, Eiseman JL. Plasma pharmacokinetics of butyrate after intravenous administration of sodium butyrate or oral administration of tributyrin or sodium butyrate to mice and rats. Cancer Chemother Pharmacol. 1999;43(6):445–53. 10.1007/s002800050922 10321503

[104] Schönfeld P, Wojtczak L. Short- and medium-chain fatty acids in energy metabolism: the cellular perspective. J Lipid Res. 2016;57(6):943–54. 10.1194/jlr.R067629 27080715 PMC4878196

[105] Boets E, Gomand S V., Deroover L, Preston T, Vermeulen K, De Preter V, et al Systemic availability and metabolism of colonic-derived short-chain fatty acids in healthy subjects: a stable isotope study. J Physiol. 2017;595(2):541–55. 10.1113/JP272613 27510655 PMC5233652

[106] Hoyles L, Snelling T, Umlai UK, Nicholson JK, Carding SR, Glen RC, et al Microbiome–host systems interactions: protective effects of propionate upon the blood–brain barrier. Microbiome. 2018;6(1):55 10.1186/s40168-018-0439-y 29562936 PMC5863458

[107] Perry RJ, Peng L, Barry NA, Cline GW, Zhang D, Cardone RL, et al Acetate mediates a microbiome-brain-B cell axis promoting metabolic syndrome. Nature. 2016;534(7606):213–7. 10.1038/nature18309 27279214 PMC4922538

[108] Paradies G, Paradies V, Ruggiero FM, Petrosillo G. Oxidative stress, cardiolipin and mitochondrial dysfunction in nonalcoholic fatty liver disease. World J Gastroenterol. 2014;20(39):14205–18. 10.3748/wjg.v20.i39.14205 25339807 PMC4202349

[109] Méndez M, Méndez-López M, López L, Aller MÁ, Arias J, Arias JL. Basal and learning task-related brain oxidative metabolism in cirrhotic rats. Brain Res Bull. 2009;78(4–5):195–201. 10.1016/j.brainresbull.2008.10.008 19015011

[110] Arias N, Méndez M, Fidalgo C, Aller MÁ, Arias J, Arias JL. Mapping metabolic brain activity in three models of hepatic encephalopathy. Int J Hypertens. 2013;2013:390872 10.1155/2013/390872 23573412 PMC3612461

[111] Kolb B. Animal models for human PFC-related disorders. Prog Brain Res. 1990;85:501–19. 10.1016/s0079-6123(08)62697-7 2094912

[112] Wang GW, Cai JX. Disconnection of the hippocampal-prefrontal cortical circuits impairs spatial working memory performance in rats. Behav Brain Res. 2006;175(2):329–36. 10.1016/j.bbr.2006.09.002 17045348

[113] Gordon JA. Oscillations and hippocampal-prefrontal synchrony. Curr Opin Neurobiol. 2011;21(3):486–91. 10.1016/j.conb.2011.02.012 21470846 PMC3138872

[114] Xia M, Liu T, Bai W, Zheng X, Tian X. Information transmission in HPC-PFC network for spatial working memory in rat. Behav Brain Res. 2019;356:170–8. 10.1016/j.bbr.2018.08.024 30170031

[115] Ito HT, Zhang SJ, Witter MP, Moser EI, Moser MB. A prefrontal-thalamo-hippocampal circuit for goal-directed spatial navigation. Nature. 2015;522(7554):50–5. 10.1038/nature14396 26017312

[116] Griffin AL. Role of the thalamic nucleus reuniens in mediating interactions between the hippocampus and medial prefrontal cortex during spatial working memory. Front Syst Neurosci. 2015;9:29 10.3389/fnsys.2015.00029 25805977 PMC4354269

[117] Radyushkin K, Anokhin K, Meyer BI, Jiang Q, Alvarez-Bolado G, Gruss P. Genetic ablation of the mammillary bodies in the Foxb1 mutant mouse leads to selective deficit of spatial working memory. Eur J Neurosci. 2005;21(1):219–29. 10.1111/j.1460-9568.2004.03844.x 15654859

[118] Jha SK, Jha NK, Kumar D, Ambasta RK, Kumar P. Linking mitochondrial dysfunction, metabolic syndrome and stress signaling in Neurodegeneration. Biochim Biophys Acta—Mol Basis Dis. 2017;1863(5):1132–46. 10.1016/j.bbadis.2016.06.015 27345267

[119] Fusco S, Pani G. Brain response to calorie restriction. Cell Mol Life Sci. 2013;70(17):3157–70. 10.1007/s00018-012-1223-y 23269433 PMC11114019

[120] Meunier CNJ, Chameau P, Fossier PM. Modulation of synaptic plasticity in the cortex needs to understand all the players. Front Synaptic Neurosci. 2017;9:2 10.3389/fnsyn.2017.00002 28203201 PMC5285384

[121] Harris KM, Weinberg RJ. Ultrastructure of synapses in the mammalian brain. Cold Spring Harb Perspect Biol. 2012;4(5):a005587 10.1101/cshperspect.a005587 22357909 PMC3331701

[122] Xing B, Li Y, Gao W-J. Norepinephrine versus dopamine and their interaction in modulating synaptic function in the prefrontal cortex. Brain Res. 2016;1641(Pt B):217–33. 10.1016/j.brainres.2016.01.005 26790349 PMC4879059

[123] Kleinridders A, Cai W, Cappellucci L, Ghazarian A, Collins WR, Vienberg SG, et al Insulin resistance in brain alters dopamine turnover and causes behavioral disorders. Proc Natl Acad Sci U S A. 2015;112(11):3463–8. 10.1073/pnas.1500877112 25733901 PMC4371978

[124] Pidoplichko VI, Dani JA. Acid-sensitive ionic channels in midbrain dopamine neurons are sensitive to ammonium, which may contribute to hyperammonemia damage. Proc Natl Acad Sci U S A. 2006;103(30):11376–80. 10.1073/pnas.0600768103 16847263 PMC1544094

[125] Ronan PJ, Gaikowski MP, Hamilton SJ, Buhl KJ, Summers CH. Ammonia causes decreased brain monoamines in fathead minnows (Pimephales promelas). Brain Res. 2007;1147(1):184–91. 17362882 10.1016/j.brainres.2007.02.015

[126] Llansola M, Montoliu C, Cauli O, Hernández-Rabaza V, Agustí A, Cabrera-Pastor A, et al Chronic hyperammonemia, glutamatergic neurotransmission and neurological alterations. Metab Brain Dis. 2013;28(2):151–4. 10.1007/s11011-012-9337-3 23010935

[127] Strandwitz P. Neurotransmitter modulation by the gut microbiota. Brain Res. 2018;1693(Pt B):128–33. 10.1016/j.brainres.2018.03.015 29903615 PMC6005194

[128] Morris G, Berk M, Carvalho A, Caso JR, Sanz Y, Walder K, et al The role of the microbial metabolites including tryptophan catabolites and short chain fatty acids in the pathophysiology of immune-inflammatory and neuroimmune disease. Mol Neurobiol. 2017;54(6):4432–51. 10.1007/s12035-016-0004-2 27349436

[129] Nestler EJ, Carlezon WA. The mesolimbic dopamine reward circuit in depression. Biol Psychiatry. 2006;59(12):1151–9. 10.1016/j.biopsych.2005.09.018 16566899

[130] Vaugeois JM, Pouhé D, Zuccaro F, Costentin J. Indirect dopamine agonists effects on despair test: Dissociation from hyperactivity. Pharmacol Biochem Behav. 1996;54(1):235–9. 10.1016/0091-3057(95)02131-0 8728563

[131] Fernández M, Mollinedo-Gajate I, Peñagarikano O. Neural circuits for social cognition- implications for autism. Neuroscience. 2018;370:148–62. 10.1016/j.neuroscience.2017.07.013 28729065

[132] Ott T, Nieder A. Dopamine and cognitive control in prefrontal cortex. Trends Cogn Sci. 2019;23(3):213–34. 10.1016/j.tics.2018.12.006 30711326

[133] Lammel S, Kook Lim B, Malenka RC. Reward and aversion in a heterogeneous midbrain dopamine system. Neuropharmacology. 2014;76(Pt B):351–9. 23578393 10.1016/j.neuropharm.2013.03.019 PMC3778102

[134] Kempadoo KA, Mosharov E V., Choi SJ, Kandel ER, Sulzer D. Dopamine release from the locus coeruleus to the dorsal hippocampus promotes spatial learning and memory. Proc Natl Acad Sci U S A. 2016;113(51):14835–40. 10.1073/pnas.1616515114 27930324 PMC5187750

[135] Kolasiewicz W, Kuter K, Nowak P, Pastuszka A, Ossowska K. Lesion of the cerebellar noradrenergic innervation enhances the harmaline-induced tremor in rats. Cerebellum. 2011;10(2):267–80. 10.1007/s12311-011-0250-9 21279489 PMC3114101

[136] Sokolov AA. The cerebellum in social cognition. Front Cell Neurosci. 2018;12(145):554–72.

[137] Locke TM, Soden ME, Miller SM, Hunker A, Knakal C, Licholai JA, et al Dopamine D1 receptor–positive neurons in the lateral nucleus of the cerebellum contribute to cognitive behavior. Biol Psychiatry. 2018;84(6):401–12. 10.1016/j.biopsych.2018.01.019 29478701 PMC6072628

[138] Li Y, South T, Han M, Chen J, Wang R, Huang XF. High-fat diet decreases tyrosine hydroxylase mRNA expression irrespective of obesity susceptibility in mice. Brain Res. 2009;1268:181–9. 10.1016/j.brainres.2009.02.075 19285041

[139] Felger JC, Treadway MT. Inflammation effects on motivation and motor activity: role of dopamine. Neuropsychopharmacology. 2017;42(1):216–41. 10.1038/npp.2016.143 27480574 PMC5143486

[140] Dandash O, Pantelis C, Fornito A. Dopamine, fronto-striato-thalamic circuits and risk for psychosis. Schizophr Res. 2016;180:48–57. 10.1016/j.schres.2016.08.020 27595552

[141] Gonzalo-Ruiz A, Alonso A, Sanz JM, Llinás RR. A dopaminergic projection to the rat mammillary nuclei demonstrated by retrograde transport of wheat germ agglutinin–horseradish peroxidase and tyrosine hydroxylase immunohistochemistry. J Comp Neurol. 1992;321(2):300–11. 10.1002/cne.903210209 1380016

[142] Del Campo N, Chamberlain SR, Sahakian BJ, Robbins TW. The roles of dopamine and noradrenaline in the pathophysiology and treatment of attention-deficit/hyperactivity disorder. Biol Psychiatry. 2011;69(12):e145–57. 10.1016/j.biopsych.2011.02.036 21550021

[143] Brenes JC, Rodríguez O, Fornaguera J. Differential effect of environment enrichment and social isolation on depressive-like behavior, spontaneous activity and serotonin and norepinephrine concentration in prefrontal cortex and ventral striatum. Pharmacol Biochem Behav. 2008;89(1):85–93. 10.1016/j.pbb.2007.11.004 18096212

[144] Felipo V, Butterworth RF. Neurobiology of ammonia. Prog Neurobiol. 2002;67(4):259–79. 12207972 10.1016/s0301-0082(02)00019-9

[145] Grippon P, le Poncin Lafitte M, Boschat M, Wang S, Faure G, Dutertre D, et al Evidence for the role of ammonia in the intracerebral transfer and metabolism of tryptophan. Hepatology. 1986;6(4):682–6. 10.1002/hep.1840060424 2426170

